# Integrating metagenome-scale metabolic models and metabolomics to explore candidate biochemical interactions in cultivated *Microcystis* phycospheres

**DOI:** 10.1093/ismeco/ycag226

**Published:** 2026-08-05

**Authors:** Juliette Audemard, Nicolas Creusot, Julie Leloup, Charlotte Duval, Sébastien Halary, Lou Mary, Mélissa Eon, Thomas Forjonel, Mohamed Mouffok, Rémy Puppo, Elodie Belmonte, Veronique Gautier, Jeanne Got, Marie Lefebvre, Gabriel V Markov, Coralie Muller, Benjamin Marie, Binta Diémé, Clémence Frioux

**Affiliations:** Inria, University of Bordeaux, INRAE, F-33400 Talence, France; Université Clermont Auvergne, INRAE, UNH, PFEM, MetaboHUB Clermont, 63000 Clermont-Ferrand, France; INRAE, UR1454 EABX, Bordeaux Metabolome, MetaboHub Bordeaux, 33612 Gazinet Cestas, France; Sorbonne Université, UPC, UPEC, CNRS, IRD, INRAE, Institut d'écologie et des sciences de l'environnement de Paris, UMR 7618-iEES Paris, F-75005 Paris, France; Muséum National d’Histoire Naturelle, UMR 7245 CNRS-MNHN, MCAM, Paris, France; Muséum National d’Histoire Naturelle, UMR 7245 CNRS-MNHN, MCAM, Paris, France; Muséum National d’Histoire Naturelle, UMR 7245 CNRS-MNHN, MCAM, Paris, France; INRAE, UR1454 EABX, Bordeaux Metabolome, MetaboHub Bordeaux, 33612 Gazinet Cestas, France; INRAE, UR1454 EABX, Bordeaux Metabolome, MetaboHub Bordeaux, 33612 Gazinet Cestas, France; Université Clermont Auvergne, INRAE, UNH, PFEM, MetaboHUB Clermont, 63000 Clermont-Ferrand, France; Muséum National d’Histoire Naturelle, UMR 7245 CNRS-MNHN, MCAM, Paris, France; Plateforme Gentyane, UMR 1095 Génétique Diversité Ecophysiologie des Céréales INRAE, UCA, Clermont-Ferrand, France; Plateforme Gentyane, UMR 1095 Génétique Diversité Ecophysiologie des Céréales INRAE, UCA, Clermont-Ferrand, France; Université de Rennes, Inria, CNRS, IRISA, Rennes, France; Université Clermont Auvergne, INRAE, UNH, PFEM, MetaboHUB Clermont, 63000 Clermont-Ferrand, France; Sorbonne Université, CNRS, Laboratoire de Biologie Intégrative des Modèles Marins, LBI2M, F-29680 Roscoff, France; Inria, University of Bordeaux, INRAE, F-33400 Talence, France; Muséum National d’Histoire Naturelle, UMR 7245 CNRS-MNHN, MCAM, Paris, France; Université Clermont Auvergne, INRAE, UNH, PFEM, MetaboHUB Clermont, 63000 Clermont-Ferrand, France; Inria, University of Bordeaux, INRAE, F-33400 Talence, France

**Keywords:** systems biology, microbial community, metabolic modeling, freshwater cyanobacteria, metabolomics, phycosphere, *Microcystis*

## Abstract

Favored by global changes, freshwater cyanobacterial harmful blooms generate major ecological, economic, and public health challenges. *Microcystis*, one of the most widespread cyanobacterial genera, grows within a phycosphere where specialized interactions with its microbiome occur, that are suspected to influence bloom appearance and its potential toxicity. Using a combination of metagenomics, metabolomics, and metabolic modeling, we characterized the culture-associated phycospheres of 12 *Microcystis* strains isolated from a French pond. The distribution of metabolic reactions within *Microcystis* was consistent with their genospecies, whereas the metabolic landscape at the community level diverged from cyanobacterial phylogeny, indicating partial functional decoupling between cyanobacteria and their associated microbiomes. Bacteria associated with the simplified phycospheres substantially expanded the metabolic repertoire of the system, while maintaining functional redundancy within and across communities. On the other hand, endometabolomic profiles were largely driven by cyanobacterial metabolic outputs, whereas exometabolomic analysis did not reveal metabolites involved in exchange processes. Metabolic modeling, together with the identification of toxic specialized metabolites produced by specific biosynthetic gene clusters, further highlighted differences in metabolic potential among phycospheres. Together, these findings deepen the understanding of *Microcystis*’ phycosphere functioning and demonstrate the value of multi-omics systems biology approaches, while suggesting that metabolic complementarity between species and across phycospheres could play a role in bloom-associated microbiome structure.

## Introduction

Freshwater cyanobacterial harmful algal blooms (cHABs) have become a globally recurring phenomenon, mainly driven by rising temperatures and increased nutrient loads resulting from anthropogenic activities [1]. These blooms, characterized by the proliferation of cyanobacteria such as *Microcystis*, pose major ecological and societal challenges through the release of large amounts of organic matter and toxic metabolites (e.g. microcystins). Such inputs disrupt ecosystem functioning, with severe consequences for biodiversity, public health, and local economies [2, 3]. Despite substantial progress in understanding the environmental drivers and dynamics of cyanobacterial blooms, these phenomena are still difficult to predict and overcome.

A particularly understudied aspect of these phenomena is the complex network of metabolic interactions within the *phycosphere*—the microscale environment surrounding phytoplankton cells and aggregates—colonized by heterotrophic bacteria. While the taxonomic composition of phycosphere-associated microbiomes varies with cyanobacterial species [4], their overall metabolic potential appears conserved, suggesting functional redundancy [5]. Microbial interactions within the phycosphere are thought to support phytoplankton growth, stress tolerance, and bloom persistence through nutrient recycling and metabolite exchange, involving commensalism and competition [6–9]. Yet, precise mechanisms and consequences of this microbial syntrophy remain poorly understood [10].

Addressing these knowledge gaps, a promising approach is to study simplified, cultivated, or synthetic microbial communities [11], as non-axenic cultures of phytoplankton host a specific but reduced microbiome [12]. In this context, genome-scale metabolic networks (GSMNs) are levers to generate hypotheses about functional roles within microbial communities, and connect genes, proteins, and biochemical reactions [13, 14]. However, applications to freshwater cyanobacterial communities remain limited, with most studies focusing on marine systems or isolated cyanobacteria [15–17]. Metabolomics complements these approaches by detecting key metabolites potentially mediating microbial interactions and refining metabolic model predictions [10, 18–21].

Here, we present a systems biology-based characterization of the taxonomic composition and co-metabolism of 12 cultivated phycospheres of *Microcystis* spp., isolated from a eutrophic freshwater pond during a bloom event. By integrating metagenomics, genome-scale metabolic modeling, and metabolomics, we investigate the metabolic capabilities of *Microcystis* strains and their associated microbiomes. This combined approach provides a functional view of the phycosphere organization, and reveals putative metabolic interactions and community-specific functions.

## Materials and methods

### Phycosphere isolation and culture

Water samples were collected in September 2021, from a suburban freshwater pond near Paris, Ile-de-France (Cergy-Pontoise, GPS coordinates lat. 49.01343 long. 2.02575), experiencing a *Microcystis* bloom (chlorophyll content *≈* 10 *μ*g.l$^{-1}$) [22].

After prefiltration through a 50 *μ*m porosity filter, 12 monoclonal *Microcystis* strains and their associated bacterial consortia were isolated by repeated single-cell or small-colony transfers on solid or liquid media under an inverted microscope (NIKON ECLIPSE TS100, Japan). Viable clones were then cultivated in 25 cm^2^ culture flasks (CORNING-FALCON) containing 10 ml of Z8 medium, used to maintain the collection under low-growth conditions. Cyanobacterial strains were maintained in the Paris Museum Collection (PMC, https://mcam.mnhn.fr/en/cyanobacteria-and-live-microalgae-470) at 18$^{\circ }$C, using white LED-powered lights providing an irradiance of 8–10 *μ*m photons/m^2^/s, with a photoperiod of 13h light/11h dark. Isolated strains and cultures were all monoclonal and non-axenic. They were then cultivated in BG11 medium, to promote rapid cyanobacterial growth in order to mimic the high-growth conditions observed during proliferation events, with a photoperiod of 16h light/8h dark and *∼*14 *μ*m photons/m^2^/s. After optimal growth, cultures were centrifuged to separately collect cell pellets and supernatants for genomic and metabolomic analyses. Two complementary extraction protocols were used for metabolomic analyses to maximize coverage (see section ‘Metabolomic data acquisition’).

### Shotgun metagenomic sequencing

DNA was extracted from lyophilized cultures (50 mg) [23] using the DNeasy PowerLyzer PowerSoil kit (Qiagen), following an adapted protocol (manufacturer’s instructions from step 5 onward) without bead beating to prevent DNA shearing and preserve long fragments for sequencing [24]. Cells were lysed with PowerBead Solution (750 *μ*l) and Solution C1 (60 *μ*l) by two 5-min incubations at 70$^{\circ }$C with 30 s of vortexing before and after each incubation. Long-read shotgun metagenomic sequencing was performed using a PacBio Revio platform (Pacific Biosciences, Menlo Park, CA, USA) at the Gentyane sequencing facility (Clermont-Ferrand, France) with detailed protocols in Supplementary Material and Methods. This sequencing technology provides High-Fidelity (HiFi) long reads, with low error rates [25, 26].

### Metagenomic assembly and binning

Reads were assembled using metaMDBG (v1.1) [27], and binned with metaBAT 2 (v2.12.1) [28]. Bin quality was assessed with CheckM2 [29] (v1.1.0). High-quality (HQ) metagenome-assembled genomes (MAGs, completeness > 90%, contamination < 5%) were taxonomically assigned using GTDB-tk (v2.4.1) [30] with the r226 GTDB release as reference [31].

MAG relative abundances and assembly statistics were calculated using Mapler (v2.0.1) [32]. Briefly, reads were aligned to contigs and bins, and MAG abundance was estimated as the proportion of reads mapping to each bin relative to the total number of reads, after trimming and merging overlapping alignments. Proportions were calculated individually for each MAG rather than grouped by bin quality categories, as proposed by default in the tool.

Contigs from non-HQ bins and unbinned contigs were concatenated to create 12 pseudogenomes (PG) containing the remaining sequences associated with each phycosphere.

### Phylogenomic analysis of *Microcystis*

The phylogenetic placement of the 12 *Microcystis* strains was inferred using a dataset of 349 genomes and previously described methods [22, 33]. Briefly, after predicting the coding sequences of 361 *Microcystis* genomes (including the 12 strains) using Prodigal (v2.6.3), an alignment of 779 concatenated single-copy genes shared by all strains in the analysis (674 919 positions) was generated by Roary (v3.13.0, default parameters except for a protein identity threshold *≥* 90%). Spurious and misaligned regions were removed using trimAl (v1.5) before phylogenetic inference was computed by RaxML (v8.2.12) with the following parameters: GTRGAMMA model, 100 bootstraps. In addition, the average nucleotide identity (ANI) was calculated between each pair of strains in the tree using fastANI (v1.33) [34] to define *genospecies* (ANI *≥*97%, [35]), and *genotypes* (ANI *≥*99%). Details about the tree construction are available in Supplementary Material and Methods.

### Gene prediction and functional annotation

Gene prediction of MAGs and PGs was performed with Prodigal (v2.6.3) [36]. Functional annotations were obtained with eggNOG-mapper (v2.1.12) [37], relying on eggNOG database (v5.0.2) [38], and diamond for sequence alignment (v2.1.9) [39].

Biosynthetic Gene Clusters (BGCs) were identified using antiSMASH (v7.0.0) [40] with default parameters. Additional targeted annotations were curated using reciprocal blast searches based on known *Microcystis* metabolic capabilities, as explained in Supplementary Material and Methods. Functions related to biogeochemical cycles were assessed using METABOLIC (v.4.0) [41].

### Genome-scale metabolic network reconstruction and comparisons

GenBank files were generated with emapper2gbk (v0.3.1) [14] from gene prediction and functional annotation. GSMNs were reconstructed from annotated genomes using Pathway Tools (v27.0) [42] via the mpwt pipeline (v0.8.6) [14]. PADMet library (v5.0.1) [43] was used to manipulate GSMNs and create SBML files [44]. This library was also used to get a description summary of the GSMNs (i.e. size, reaction presence, and pathway completeness), and for the curation and integration of missing reactions. For the latter, we first used the double-blast procedure described in Supplementary Material and Methods for protein annotation, then drafted the reactions catalyzed by the identified proteins for integration into the corresponding GSMNs with PADMet. Curation is further detailed in Supplementary Material and Methods.

Regarding the PGs, a single GSMN was reconstructed from concatenated unbinned contigs and low-quality bins per phycosphere, retaining only gene-associated reactions.

Details of GSMNs comparison are described in Supplementary and Methods. Metabolome-to-GSMN mapping were performed using MetaNetMap (v1.0.1) [45] (details in Supplementary Material and Methods).

### Metabolic modeling

Community metabolic potential was assessed using the Metage2Metabo (M2M, v1.6.1) [14] pipeline integrating MisCoTo (v3.2.0) [46], and MeneTools (v3.4.0) [43]. Those rely on network expansion, a Boolean abstraction of metabolic producibility. They assess reachability of metabolites in the GSMN from available *seed* metabolites [47] corresponding to BG11 medium composition (Supplementary Table S1), while accounting from complementarity of metabolic functions within the microbial community (details in Supplementary Material and Methods).

Two modeling scenarios were explored: (i) intra-phycosphere metabolic complementarity under laboratory culture conditions, and (ii) inter-phycosphere metabolic complementarity simulating pond-like environmental conditions (details in Supplementary Material and Methods).

### Metabolomic data acquisition

Intracellular metabolites from the 12 phycosphere cultures were collected by centrifugation (3,200 x g 15$^{\circ }$C, 10 min), lyophilized and extracted using monophasic protocol (MP) [48], mainly dedicated to capturing specialized metabolites, and biphasic protocols (BP) [49], to maximize metabolome coverage. Each extract was injected in triplicate. Details about the MP and BP protocols are provided in Supplementary Material and Methods. The metabolomic data have been deposited in the MetaboLights [50] repository with the study identifier MTBLS13909.

### Metabolomic annotation and chemometrics

Feature extraction and annotation of metabolomic signals were performed according to two different workflows. First, for MP extracts, the mass spectrometry (MS) data were processed using MetaboScape (v4.0) software (Bruker, Bremen, Germany) in order to generate a data matrix containing semi-quantification results for each analyte in all analyzed samples (see details in Supplementary Material and Methods). Then, MS-DIAL (v5.25) [51] and MS-cleanR were used to process and clean both MP and BP data and interrogate FragHub spectral libraries [52]. Prior chemometrics, technical replicates were averaged to keep one signal per metabolite for each phycosphere. Data tables from MP and BP protocols were concatenated and feature intensity was normalized, transformed, and scaled (i.e. sum normalization, cube root transformation, and pareto scaling). A principal component analysis (PCA) and a hierarchical clustering analysis (HCA) were performed and annotated metabolites were summarized according to chemical classes (NPC classification [53]). SIRIUS software (v6.1) [54] was used to interrogate structural libraries (SIRIUS database and cyanoMetDB [55]). Output files from MS-DIAL and SIRIUS were further imported into MS-Net [56] in order to suppress analytical redundancy, provide annotation confidence levels and classification (NPclassifier [53]). Annotation workflow parameters are provided in Supplementary Material and Methods. Following MS-Net, spectral library matches were stratified as level 1 (spectral cosine > 0.95 and $\Delta RT < 0.2\,\textrm{min}$), level 2a (cosine > 0.85) or 2b (0.7 *≤* cosine *≤* 0.85). *In silico* candidates were supplemented with taxonomic information by querying COCONUT 2.0 [57] using InChIKey-based matching. Among the candidates per feature, those matching taxonomic criteria (identified in bacterial genera or in *Microcystis*) were classified at level 3a, and the remaining *in silico* matches stayed at level 3b. Spectral library matches with cosine < 0.7 or significant precursor mass errors were classified as level 4 (MS/MS analogues).

### Statistical analysis and data visualization

Data analysis was handled with Python (v.3.12.3), Pandas (v.2.2.2), Numpy (v.2.1.0), or Scikit-learn (v.1.6.1), as well as R (v.4.4.1) package Vegan (v.2.6_6.1). Visualization was generated using Python Matplotlib package (v.3.9.2), and R package Dendextend (v.1.17.1) for tanglegrams.

Phycosphere taxonomic trees were constructed in newick format using GTDB-tk results [58], and displayed with the iTOL tree display and annotation tool [59]. GSMNs similarity across phycospheres were assessed by performing KR Clarke analysis of similarity [60], and visualized with metric multidimensional scaling (metric MDS). Details on the comparison are given in Supplementary Material and Methods.

## Results

### Phylogenomic and potential metabolism of the 12 *Microcystis* strains

A total of 9 006 345 HiFi metagenomic reads (67.9 Gbp) were obtained, ranging from 516 628 to 1 021 017 per phycosphere community, with median contig lengths between 5973 and 7951 bp. One complete *Microcystis* genome (single contig) was reconstructed from each non-axenic culture, with an average size of 5.3 Mbp.

#### Phylogenomics of *Microcystis* strains

**Figure 1:**
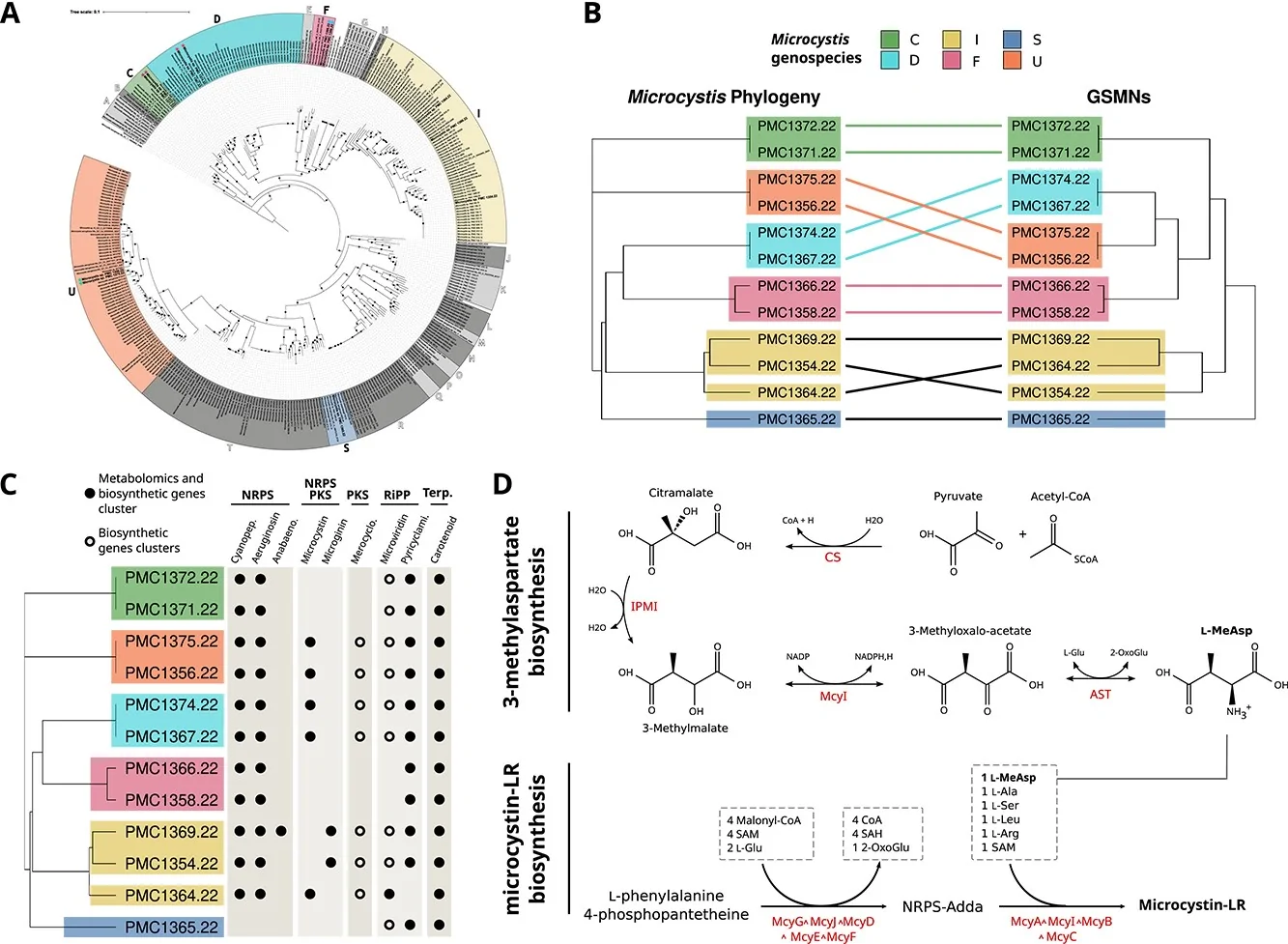
Reconstruction of metabolism for the 12 *Microcystis* strains. (A) Maximum-likelihood phylogenomic tree of *Microcystis* species. The boxes group together strains from the same genospecies, labelled from A to U. The coloured ones (C, D, F, I, S, and U) correspond to the genospecies of the surveyed strains (shown in bold). The stars associated with the labels indicate that the associated strains belong to the same genotype. Tree branches supported by bootstrap values over 60% are indicated by a black circle. The tree is rooted at *M. aeruginosa* Ma_SC_T_19800800_S464, as determined in previous studies [35]. (B) Tanglegram of *Microcystis* ANI phylogenetic tree on the left, and of the hierarchical clustering of GSMN’s reactions presence matrix on the right. Hierarchical clustering was conducted using Jaccard distance and complete-linkage clustering. Species on branches that are coherent between the two dendrogram are connected by a coloured link. (C) Specialized metabolism of *Microcystis* strains. On the left, the phylogenetic tree of the *12 Microcystis* strains. On the right, a matrix of the specialized metabolites biosynthetic genes clusters (BGCs) presence in genomes, and the identification of metabolites in the phycosphere based on metabolomic data from multiplexed LC-HRMS/MS analyses (Supplementary Material). (D) Microcystin-LR (MC-LR) and 3-methylaspartate biosynthesis reactions manually added to *mcy*-bearing *Microcystis* GSMNs, according to the literature, and identified genes in the 12 *Microcystis* genomes. At the top, l-MeAsp putative biosynthesis. At the bottom, the reactions of the MC-LR biosynthesis integrated into the GSMNs, represented as a lumped reaction. Abbreviations: Anabeno., anabenopeptin; Cyanopep., cyanopeptolin; Merocyclo., merocyclophane; NRPS, non-ribosomal peptides synthase; PKS, polyketide synthase; Pyricyclami., pyricylamide; RiPP, ribosomally synthesized and post-translationally modified peptides; Terp., terpenoids; 2-OxoGlu, 2-oxoglutarate; Adda, (2S,3S,4E,6E,8S,9S)-3-amino-9-methoxy-2,6,8-trimethyl-10-phenyl-4,6-decadienoic acid, AST: aspartate transaminase; CS, citramalate synthase; l-MeAsp, l-methylaspartate; IPMI, isopropylmalate isomerase acid.

*Microcystis* phylogeny was inferred from a concatenated alignment of 779 single-copy genes (674 919 aa positions) shared by 361 strains. The 12 strains captured local *Microcystis* diversity and were distributed across the phylogenomic tree, with *Microcystis* sp. PMC1371.22 and *Microcystis* sp. PMC1372.22 closest to the root (Fig. 1A). Using ANI threshold *≥* 97% [35], 21 genospecies (A-U) gathering multiple strains were identified encompassing 352 strains. The 12 strains belonged to six distinct genospecies (C, D, F, I, S, and U). All strains within a genospecies belong to a single genotype (ANI *≥* 99%)), except for genospecies I, which three strains belong to three distinct genotypes.

#### Potential functional metabolism of *Microcystis*

GSMNs reconstructed for the 12 *Microcystis* genomes were highly similar (from 1780 to 1823 reactions; Table 1), consistent with their phylogenetic proximity (Fig. 1A, Supplementary Fig. S1). The quality of GSMN reconstruction was validated by the congruence between metabolic distances across strains and GSMN contents (Fig. 1B). This suggests that the reconstruction captured clade-specific metabolic features. *Microcystis* GSMNs were identical within genospecies D (PMC1374.22 and PMC1367.22) and U (PMC1375.22 and PMC1356.22), although undetected differences may exist due to annotation limits, and the prevalence of unknown cyanobacterial genes [61].

**Table 1:** Description of the 12 phycosphere metagenomes and their reconstructed GSMNs. For each community, a distinction is made between *Microcystis*, its associated bacteria, and the pseudogenome (PG) reconstructed from the remaining contigs of the assembly. PG’s reactions introduced in the phycosphere is the percentage of reactions of the pseudogenome’s metabolic network that are not in any of the other phycosphere’s members GSMN. On the far left column, the *Microcystis* genospecies is indicated.

		Cyanobacteria			Associated bacteria (average data)				Pseudogenome		
Phycosphere		Genes	Reactions	Reactions with genes association (%)	HQ MAGs	Genes	Reactions	Reactions with genes association (%)	Genes	Reactions	Added value (%)
**C**	**Phyco1371$^{\textrm{a}}$**	5251	1790	68.1	8	3498.4 *±* 893.7	2029.0 *±* 287.8	67.3 *±* 0.8	9677	1937	12.1
	**Phyco1372$^{\textrm{a}}$**	5256	1791	68.1	10	3538.6 *±* 859.2	2068.1 *±* 278.4	67.4 *±* 0.9	19128	2359	9.0
**D**	**Phyco1367**	5501	1792	68.2	7	3622.9 *±* 1179.2	1971.6 *±* 267.7	67.5 *±* 1.7	**567**	**183**	4.4
	**Phyco1374$^{\textrm{a}}$**	5491	1792	68.2	8	**3968.5 *±* 1130.1**	2075.4 *±* 318.4	67.9 *±* 2.0	15188	2123	5.0
**F**	**Phyco1358**	4897	1784	68.3	**5**	3949.2 *±* 660.2	2014.0 *±* 216.5	67.1 *±* 1.4	15773	2121	12.8
	**Phyco1366**	4944	**1780**	68.2	**5**	3757.2 *±* 596.3	2045.2 *±* 204.1	67.9 *±* 2.2	11191	1960	14.5
**I**	**Phyco1354**	4909	**1823**	**67.7**	12	3718.8 *±* 1074.1	1971.9 *±* 273.7	67.8 *±* 1.4	15702	2054	**4.3**
	**Phyco1364**	**4864**	1796	68.5	7	3760.0 *±* 948.2	**1941.6 *±* 258.5**	67.5 *±* 0.5	15255	2229	11.8
	**Phyco1369**	5323	1795	**68.9**	7	3620.4 *±* 737.1	1960.9 *±* 212.8	67.2 *±* 0.8	13385	2212	13.4
**S**	**Phyco1365$^{\textrm{a}}$**	4627	1795	68.6	**13**	3967.8 *±* 1167.2	**2083.4 *±* 263.7**	**68.0 *±* 1.6**	14651	2265	5.7
**U**	**Phyco1356$^{\textrm{a}}$**	**5784**	1795	67.8	8	**3419.4 *±* 601.4**	1948.4 *±* 191.4	67.1 *±* 1.4	11426	2036	9.9
	**Phyco1375$^{\textrm{a}}$**	5774	1795	67.8	6	3483.0 *±* 807.4	1943.7 *±* 284.1	**67.0 *±* 1.1**	**33414**	**2839**	**23.5**

Bold: minimum and maximum values. $^{\textrm{a}}$: Phycosphere remaining colonial under laboratory-culture conditions.

Specialized metabolism (SM), largely encoded by BGCs, was surveyed across genomes (Fig. 1C, Supplementary File 1). SM refers to the biosynthesis of metabolites that are not essential for basic cellular growth but provide ecological functions, such as defense, competition, or signalling [62–64]. BGC content was strictly conserved within genotypes and more loosely conserved within genospecies [65], as illustrated by genospecies I, which exhibits greater genetic divergence (97% < ANI < 99%) among its three strains belonging to distinct genotypes. Only five strains from genospecies U, D, and I carried microcystin-associated BGCs while carotenoid-associated BGCs were detected in all strains. *Microcystis* PMC1365.22 (genospecies S) lacked most BGCs. Most SM products were experimentally detected in the intracellular metabolomes, except microviridin, which was detected only in PMC1364.22, potentially due to sequence variability and annotation limitations.

SM-associated reactions were absent from GSMNs and the MetaCyc database [66] highlighting the limited representation of SM in GSMN reconstruction as previously established [67]. To address this, the microcystin-LR (MC-LR) biosynthetic pathway was manually curated (Fig. 1D), integrating literature (Suppplementary Table S8) and BGC annotations. Overall, *Microcystis* strains shared a conserved core metabolism with phylogenetically structured SM diversity, including variation in pigment and toxin production.

### Taxonomy and functions of the associated microbiome

#### Taxonomic composition of phycosphere’s associated bacteria

**Figure 2:**
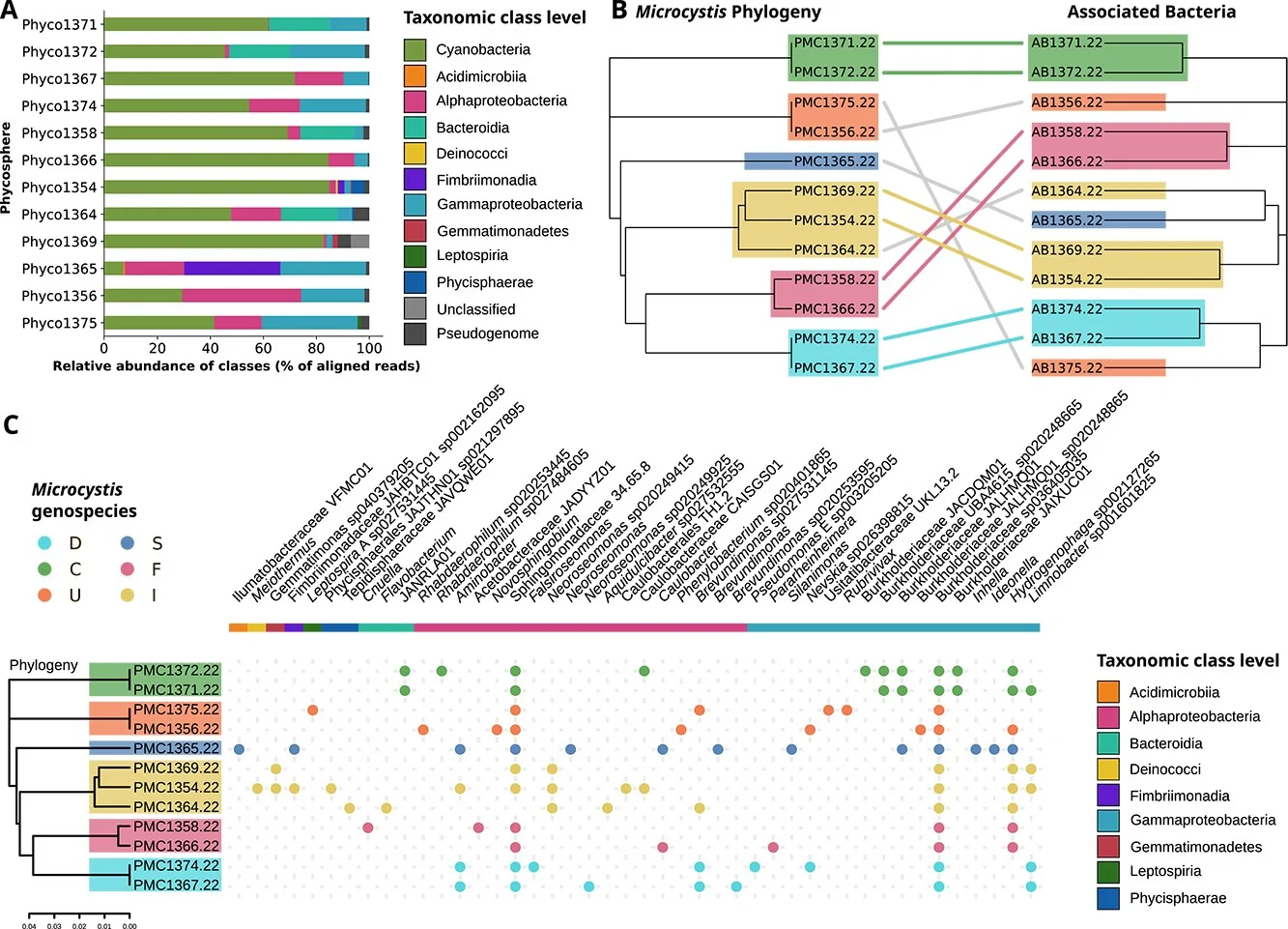
Metagenomic assembly of the 12 phycospheres. (A) Class taxonomy level and relative abundance of the associated bacteria based on metagenomic read mapping. (B) Relationships between *Microcystis* strain phylogenomy and the taxonomic composition of the respective associated bacteria according to hierarchical classification. (C) Heatmap of the bacterial MAGs occurrence within the 12 phycospheres.

Ninety-six HQ MAGs (5–13 per phycosphere) of associated bacteria (AB) were recovered, including 62 MAGs nearly, or fully complete and assembled in a unique contig (Supplementary File 1). HQ MAGs accounted for *>90%* of read abundance in most phycospheres, with *Microcystis* genomes showing the highest coverage (82X to 1115X) consistent with culture conditions favoring cyanobacterial growth (Supplementary Fig. S2). Cyanobacterial relative abundance varied among phycospheres, with *Phyco1365* (associated with *Microcystis* PMC1365.22), *Phyco1356* (associated with PMC1356.22) and *Phyco1375* (PMC1375.22) displaying lower dominance, suggesting distinct community structures (Fig. 2A, Supplementary Fig. S2). Unbinned contigs and lower-quality MAGs were merged into phycosphere-specific pseudogenomes (PGs) to capture residual functional diversity from low-abundant unassembled or unbinned populations.

Taxonomic assignment of MAGs (Fig. 2A, Supplementary Fig. S3) revealed that most AB belonged to *Alphaproteobacteria, Gammaproteobacteria*, or *Bacteroidia*. Species-level composition of phycospheres (Fig. 2C) often mirrored *Microcystis* phylogeny, with several MAGs associated with specific genospecies: e.g. *Neoroseomonas sp020249415* with genospecies I, *JANRLA01* (Bacteroidia) and *Burkholderiaceae UBA4615_sp020248665* with genospecies C. In contrast, *Burkholderiaceae sp036405035* and *Sphingomonadaceae 34.65.8* were nearly ubiquitous, indicating stable associations across genospecies. *Fimbriimonadia* were enriched in *Phyco1365* which showed lower cyanobacterial dominance, and also found in smaller proportion in *Phyco1354*.

Phylogenetically structured associations [17] were assessed by comparing the phylogeny of *Microcystis* with the dendrogram based on the distances between the 12 associated microbiomes (Fig. 2B). The strongest congruence occurred at species and genus levels (Normalized Robinson-Foulds (NRF) metric [68]: 0.55), with clustering by genospecies for several genotypes (D, F, C, and I (2/3 strains)). The dendrogram of genospecies U and its AB was not obviously consistent between the two distinct strains, according to slight AB community variation. Higher taxonomic ranks (family and order) showed weaker strains specificity and congruence (NRF: 0.88 and 0.77 resp. Supplementary Fig. S4), indicating that phylogenetically structured associations may primarily operate at fine taxonomic resolution, that would imply even more pronounced metabolic specificities.

#### Metabolic potential of associated bacteria in culture-selected phycospheres

GSMNs of AB and PGs were heterogeneous and generally larger than *Microcystis* networks, averaging 2010 reactions (Table 1). Some PGs yielded small networks (e.g. *Phyco1367*), whereas others were larger but largely redundant with the rest of the phycosphere. PGs of *Phyco1366* and *Phyco1375* contained many genes and contributed the most additional unique reactions to the community, with, respectively, 14.5% and 23.5% of unique reactions, suggesting the presence of low-abundance—uncaptured—populations in those phycospheres with distinct metabolic capabilities.

**Figure 3:**
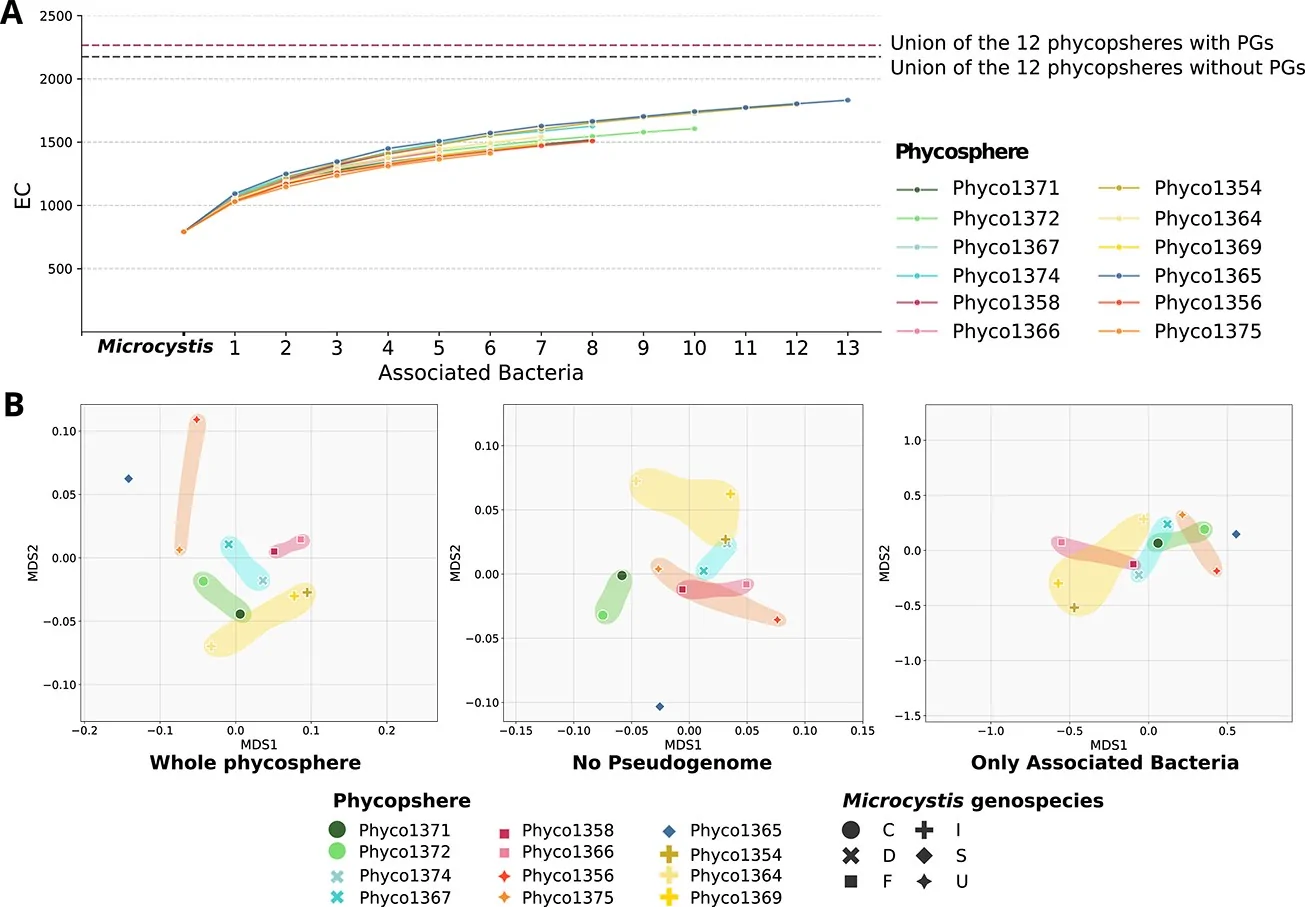
Metabolic complementarity within phycospheres. (A) Rarefaction curve of EC numbers annotated in phycosphere metagenomes. Rarefaction curves represent the accumulation of non-redundant EC numbers as a function of the number of sampled associated bacteria, based on 100 sampling iterations. The starting point is *Microcystis* EC numbers. PGs are excluded from the sampling of phycosphere’s individual curves. (B) Metric multidimensional scaling of the GSMNs reactions content, weighted by species abundance and grouped by phycosphere. From left to right; for the whole phycospheres, without the pseudogenome, and with only the associated bacteria. Shaded areas show phycospheres associated with *Microcystis* strains belonging to the same genospecies. Abbreviations: EC, Enzyme Commission number; PG, pseudogenome.

To further investigate functional redundancy and complementarity within phycosphere communities, we performed a rarefaction analysis of the number of enzyme commissions (EC) and other functional annotations in *Microcystis* and AB genomes, excluding PGs (Fig. 3A, Supplementary Fig. S5). The absence of a plateau indicates that even in these small cultivated phycospheres, each member likely contributes unique functions. However, the curve inflected beyond five members, suggesting diminishing functional novelty with increasing community size. The union of all reconstructed phycosphere genomes set an upper limit of *∼* 300 additional EC numbers, whereas PGs contributed *∼* 100 EC numbers absent from other genomes. Analysis of genes related to biogeochemical cycles (Supplementary Material) revealed clear functional partitioning between community members. Cyanobacteria and AB differed in nitrogen metabolism, whereas heterotrophic bacteria encoded pathways for the degradation of complex carbon substrates.

We next compared metabolic reaction repertoires across phycospheres using three configurations: all members including PGs, all members excluding PGs, and AB only (Fig. 3B). Complete phycospheres showed a significant but moderate clustering according to *Microcystis* genospecies (KR Clarke analysis of similarity [60], *R=0.539*, *P*-value *=.007*). This pattern persisted when PGs were excluded (*R=0.554*, *P*-value *=.005*) but weakened when only AB were considered (*R=0.317*, *P*-value *=.084*). In all analyses, phycospheres associated with genospecies I displayed the highest functional diversity.

### Meta-metabolomes of the phycospheres

**Figure 4:**
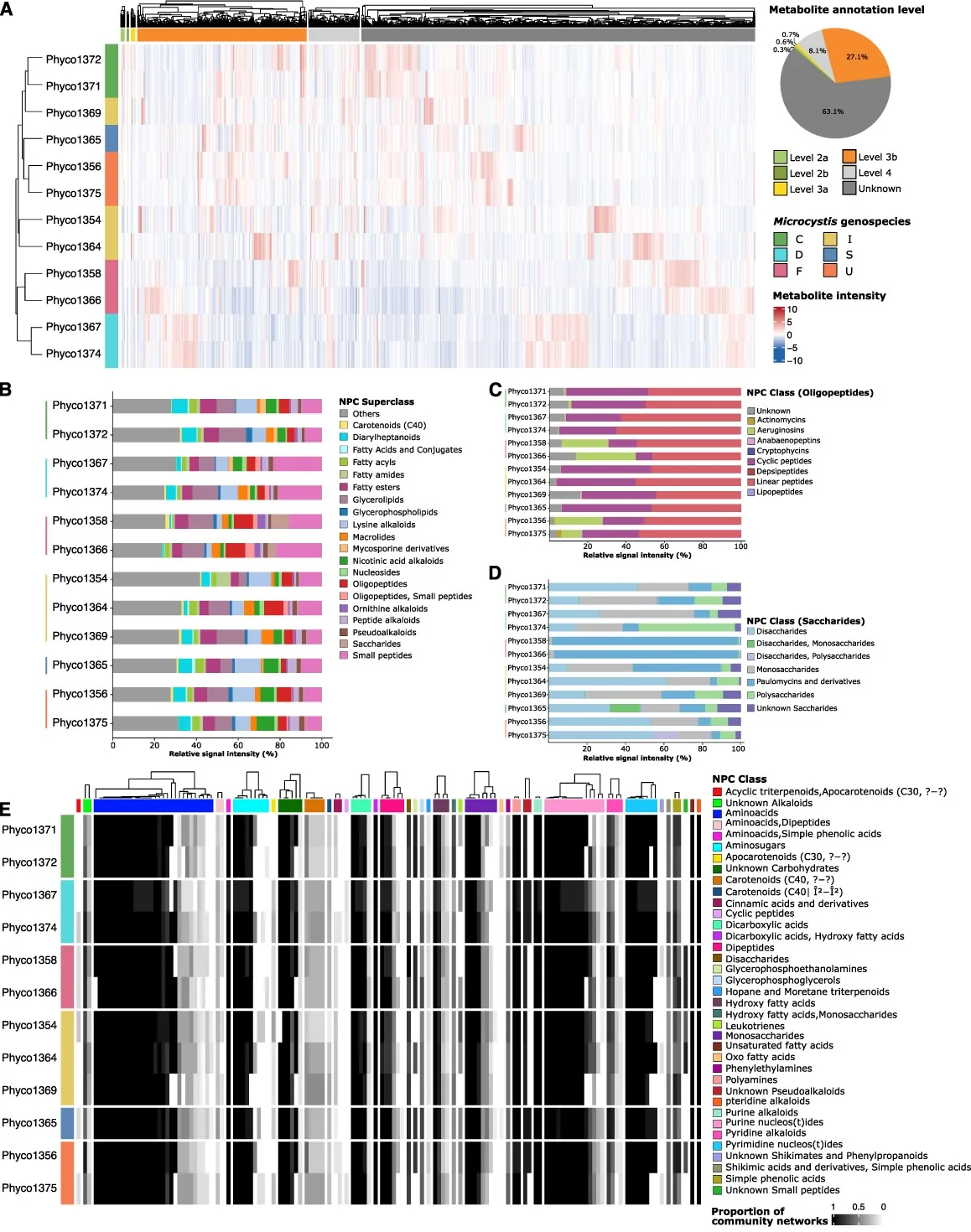
Metabolomic analysis of the 12 phycospheres. (A) Hierarchical clustering analysis of the phycospheres based on -transformed endometabolome signals. For each phycosphere, triplicates were averaged to merge the results from the different extractions and to obtain one set of transformed signals per community. Metabolite annotation was conducted as previously described, and evaluated with levels assigned as described in Methods. Hierarchical clustering was based on euclidean distance and complete linkage method. (B) Barplot depicting the relative abundance of L2 and L3 annotated metabolomic signals in phycospheres stratified by NP Classifier superclasses. Only the 20 most abundant superclasses are depicted, with the others aggregated into *Others*. (C) Focus on the annotated metabolomic signals classified as the ‘Oligopeptide’ superclass (relative abundance). The 20 most abundant classes are depicted, with the others aggregated into *Others*. Cyclic peptides include microcystins, additional toxins, and other specialized metabolites, such as aeruginosins, or cyanopeptolins. (D) Focus on the annotated metabolomic signals classified as the ‘Saccharides’ superclass. Relative abundance of saccharides classes in the 12 phycospheres. (E) Heatmap depicting the mapping of L2-L3 annotated metabolomic signals onto the GSMNs of all phycosphere members. Heatmap values represent the proportion of GSMNs per phycosphere that contain the related metabolite. Identification in a GSMN means that the metabolite is a substrate or a product of a metabolic reaction. Metabolites are stratified by their NP-Classifier classes. Cyclic peptides correspond to microcystin-LR. Carotenoids include echinenone, canthaxanthin, zeaxanthin, adonixanthin, and diketospirilloxanthin. Abbreviations: NPC, NP Classifier.

The merged endometabolome features (from both MP and BP extractions; Supplementary Fig. S6 and Fig. 4A) of the 12 phycospheres comprised 20 931 features: 12 608 with only MS1 and 8323 with MS2 spectra. Among these, 0.6% (135 features) and 0.3% (63 features) were annotated into levels 2a and 2b, respectively. Including *in silico* candidates (level 3a, 144 features and 3b, 5674 features), up to around 31% of features were putatively annotated. Both HCA (Fig. 4A) and PCA (Supplementary Fig. S7) revealed clustering of phycospheres according to *Microcystis* genospecies, consistent with a massive cyanobacteria-driven metabolite production and likely influenced by the high *Microcystis* biomass dominating the cultures, which is also fully congruent with our metagenomic observations considering the more than several hundred times higher biovolume of *Microcystis* cells compared with heterotrophic AB (Fig. 2A). Only slight deviations were observed, with *Phyco1369* diverging from genospecies I and *Phyco1365* showing a metabolomic profile closer to genospecies U (Fig. 4A). Annotated metabolites (6016 features with L2 or L3 confidence) were categorized by NPC classification (Supplementary Fig. S8) and the 20 most represented superclasses were highly similar across phycospheres (Fig. 4B). Although some of the isolated *Microcystis* strains in this study still exhibited colonial organization under laboratory culture conditions (Table 1), we were not able to detect specific metabolite signatures indicating that these strains produced different types or amounts of annotated metabolites (Fig. 4; Supplementary Figs S7 and S8). In particular, similar saccharide profiles were observed in strains displaying colonial structures—likely supported by mucilage embedding the cells in the so-called colonies—and in non-colonial strains. Only 144 of the annotated features could be specifically linked to taxa detected by metagenomics, mainly *Microcystis, Pseudomonas*, and *Burkholderiaceae* (details, including putative ecological roles and compound identities, in Supplementary Material, Supplementary Fig. S9).

The oligopeptide NPC_superclass (14% of annotated compounds), showed strong variability among phycospheres, with genospecies-level similarities (Fig. 4C). Actinomycins (level 3b) and linear lipopeptides were specific to genospecies U and C, respectively, while microcin SF608 (level 3a), likely produced by AB, was enriched in genospecies U. Thirty-six variants of microcystins were putatively annotated (levels 2-3), including microcystin-RR and MC-LR; they were most abundant in *Phyco1374* and *Phyco1367* (genospecies D), *Phyco1375* and *Phyco1356* (genospecies U), and especially in *Phyco1364*, matching BGC-based predictions (Fig. 1C). Additionally, a few low-intensity putative microcystins were detected in strains lacking microcystin BGCs. These are likely false positives arising from limitations of automated metabolite annotation, as certain micropeptins/cyanopeptilins share structural features with microcystins but can be manually distinguished based on the presence of specific diagnostic ions.

Saccharides, given their potential role in cross-feeding [69], were examined and varied by genospecies (Fig. 4D); e.g. paulomycins (level 3b) were most abundant in genospecies F phycospheres, and some monosaccharides were detected exclusively in *Phyco1365*, the sole representative of genospecies S (Figs 1C and 2A). No relevant metabolites were detected in the exometabolome beyond culture medium BG11 components, suggesting rapid consumption of extracellular components by heterotroph AB or reduced interactions under optimal growth conditions.

Mapping metabolomic features to phycosphere GSMNs yielded only 133 matches (Fig. 4E). Mapped metabolites were mainly amino acids, amino sugars, dipeptides, purines, pyrimidines, and nucleosides, with variable distribution across GSMNs. Notably, carotenoids were mapped only to GSMNs of AB, including PGs, which may correspond to a false negative for *Microcystis* whose carotenoid biosynthetic genes remain poorly characterized and further automatically annotated.

### Modeling the intra- and inter-phycosphere metabolic complementarity

**Figure 5:**
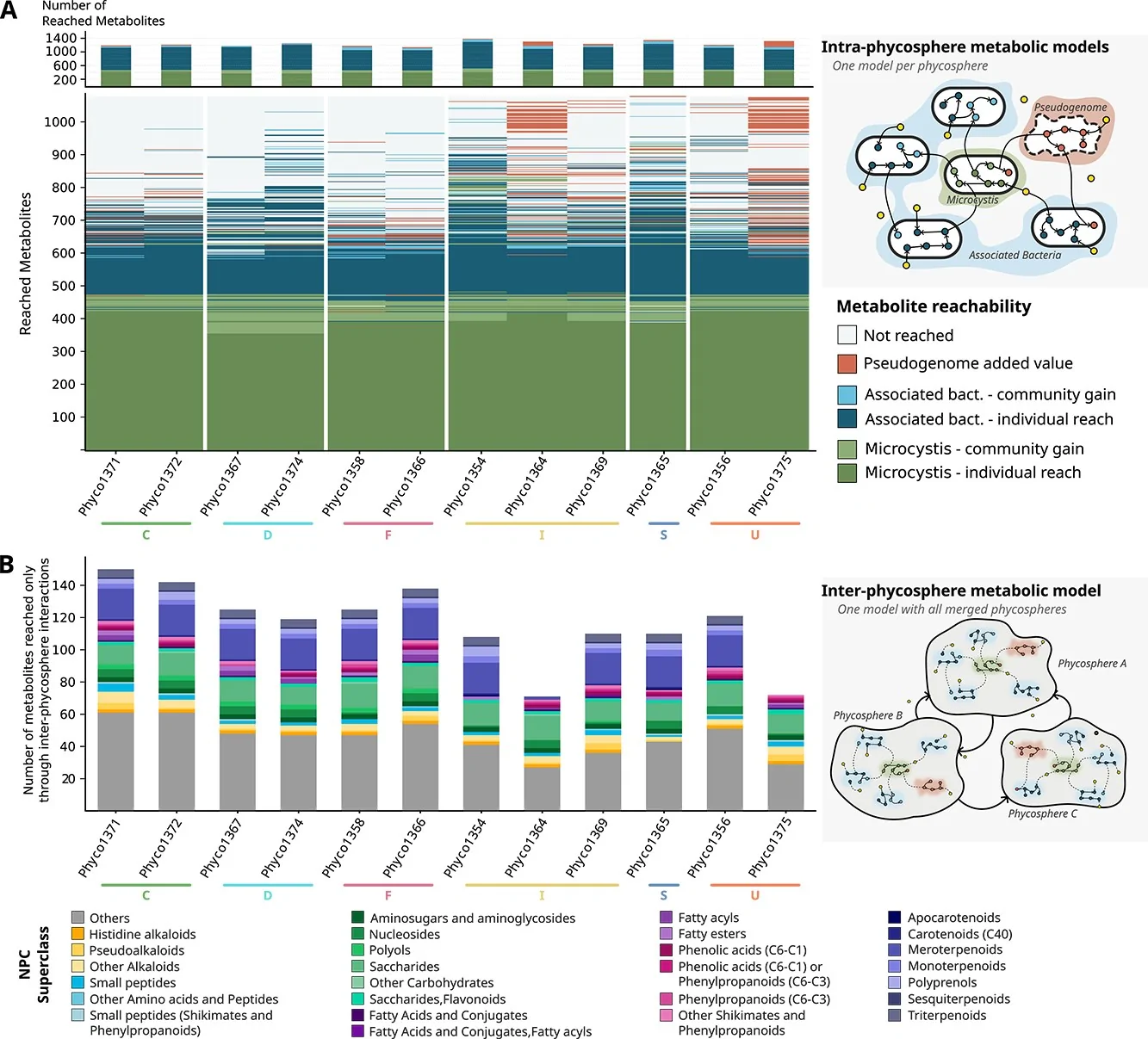
Modeling the metabolic complementarity intra- and inter-phycospheres. (A) Heatmap of metabolites putatively reached by each phycosphere according to metabolic capabilities modeling, grouped by genospecies (C, D, F, I, S, and U). Producers of reachable metabolites were grouped to distinguish the roles of *Microcystis*, associated bacteria (AB), and pseudogenomes (PGs), as well as to assess the impact of interactions within and between these groups. Each row is a metabolite, preferentially shown as reached by the cyanobacteria, when it is also reached by one of the AB. Non-overlapping reachability is presented in Supplementary Fig. 9. ‘Individual reach’, metabolites predicted to be reached by at least one community member in the phycosphere; ‘Added value’, metabolites predicted to be reached only through metabolite exchange; ‘not reached’, metabolites not reached in modeling or absent from GSMNs. The upper barplot enumerates the (possibly overlapping) metabolites reached in each of the observed situation (by *Microcystis*, AB, through the PG). (B) Classification of inter-phycosphere added value from simulation results. For each phycosphere, metabolites that are predicted to be reached by one of its members only through phycosphere-to-phycosphere exchanges are classified according to their NPC superclass level.

To investigate metabolic complementarity within and across phycospheres, we simulated metabolite production for each GSMN under BG11 medium conditions, i.e. we identified the metabolites of the GSMNs that could be reached from these nutrients. Predictions distinguished metabolites produced individually from those requiring mutualistic interactions in the model. We tested two scenarios: (i) separate simulations for each phycosphere to assess within-community complementarity, and (ii) a merged network simulation to survey putative larger-scale interactions between phycospheres (Fig. 5).

Intra-phycosphere metabolic potentials and interactions are shown in Fig. 5A. Under the simulated conditions, *Microcystis* could access a core of over 400 metabolites, largely autonomously. Additional metabolites became accessible only through metabolic interactions with AB and PGs, with variability among phycospheres. Differences among cyanobacterial GSMNs were largely offset by the community composition (Fig. 1 and Table 1). In contrast, AB metabolic potential varied despite a core of 510 shared metabolites and did not align cyanobacterial genospecies (analysis of similarity [60], *R=0.355, P=.07*). PGs unlocked additional pathways, contributing *∼*25 unique metabolites per phycosphere. *Phyco1364* and *Phyco1375* shared unique predicted cyanobacterial metabolites, mainly terpenoids. Comparisons between model predictions and metabolomics are detailed in Supplementary Material.

Inter-phycosphere simulations, including PGs, predicted reachability of *∼*100 additional metabolites per community (Fig. 5B), such as fatty acids, shikimates, phenylpropanoids, carbohydrates, and terpenoids. Notably, terpenoid functions from *Phyco1364* and *Phyco1375* spread to other communities, suggesting putative transferable metabolic benefits.

Together, these results indicate that metabolic complementarity could enhance metabolite accessibility both within and between phycospheres and is shaped by phycosphere composition.

## Discussion

### *Microcystis* metabolism reflects genospecies structure

*Microcystis* genomes are highly plastic [61], with non-ribosomal peptide synthase and polyketide synthase gene clusters accounting for 5% of the genome [70], and *∼*240 natural products classified as specialized metabolites through genomics [71, 72]. Given their high versatility, capturing metabolic variation across genospecies is therefore essential. Our GSMN reconstructions revealed a strong consistency between metabolic reaction content and genospecies phylogeny. BGC repertoires were similarly conserved within genospecies, except for genospecies I, which displayed greater heterogeneity. Metabolomic analyses further confirmed the production of multiple microcystin variants by *Microcystis*. However, their biosynthetic pathways were largely absent from GSMNs due to incomplete database representation. Manual curation of the MC-LR pathway in GSMNs underscores the substantial efforts still required to incorporate SM into genome-scale models and could benefit from existing efforts in BGC description [67, 73].

### Phycospheres harbur a reduced taxonomic diversity driven by *Microcystis* phylogeny

The reduced taxonomic diversity observed in phycospheres is consistent with culture conditions [12, 74, 75], and reflects selective pressure favoring *Microcystis* growth thus prioritizing AB that are essential for it, or at least non-antagonistic [74, 76]. It was previously reported that bacterial communities in the phycosphere are shaped by the genotypic characteristics of *Microcystis* [17]. Despite the limited dataset, this observation implies that bacterial partners conserved through cultivation process may present specific selective added-value that directly depends on *Microcystis* genospecies. The molecular mechanisms behind, potentially mediated by metabolic complementarity or selective processes, remain however unknown so far.

Dominant taxa, including Caulobacterales and Burkholderiales, are frequently reported in cyanobacterial phycospheres in both environmental and laboratory studies. The ubiquitous presence of *Hydrogenophaga* sp. aligns with its recurrent association with *Microcystis*, in natural environments and laboratory cultures [12, 74, 77], while *Sphingomonadales*, known for microcystin tolerance and degradation [78, 79], were systematically detected. While metabolomic and BGC analyses confirmed microcystin production within the phycospheres, the absence of known microcystin degradation genes (*mlr*) suggests that tolerance, rather than active degradation, may be the main adaptation mechanism of these bacterial partners, although yet undescribed degradation pathways cannot be excluded.

### Functional complementarity within the phycosphere, but a cyanobacteria-driven metabolome

Comparative functional analyses and GSMNs revealed substantial diversity and metabolic complementarity among phycosphere members. Notably, PGs contributed *∼*23% unique metabolic reactions to phycosphere, highlighting the underestimated role of low-abundance community members. While AB functions alone did not reflect *Microcystis* genospecies, genospecies-specific signals emerged when considering the full phycosphere. *Microcystis* strains likely influence their communities via their own metabolism [17] but the lack of a clear signal at the global AB genomic level suggests that community shaping depends more on specific metabolic functions or environmental responses. Functional complementarity included nitrate and nitrite reduction by AB, potentially supporting redox balance and nutrient recycling [75, 77]. In contrast, thiosulfate oxidation genes were absent from *Microcystis* but present in *Limnobacter* spp. in half of the phycospheres, consistent with prior detection during bloom events [77]. These results are consistent with the findings of Huang *et al*. [80], which suggest the existence of metabolic cooperation within the phycosphere of *Microcystis* colonies.

Yet, metabolism captured by metagenomics and annotation likely reflects only a fraction of the total metabolic landscape, as illustrated by the limited fraction of metabolomic features that could be directly linked to GSMNs, highlighting opportunities for improvement [81]. The discrepancy between annotated metabolites and those successfully mapped is a common issue in metabolomics studies and can arise from various factors, including difficulties in converting metabolite names and database identifiers across resources [82]. However, our analysis also revealed a lack of reactions associated with secondary metabolism in the GSMNs. This finding confirms that automatically reconstructed GSMNs are incomplete with respect to SM [67], which may further contribute to the low mapping rate observed.

Metabolomic annotations captured only a small fraction of all detected signals, with fewer than 2% annotated at level 2 and most assigned *in silico*. The absence of annotated exometabolites prevented the formal inference of metabolite exchange, possibly due to optimal growth conditions or rapid metabolite turnover. To better identify potential metabolic interactions, future analyses could investigate phycosphere dynamics over time through multiple sampling points, or subject the cultures to stress conditions such as nutrient limitation, which may enhance metabolite exchange between partners. Nonetheless, endometabolome analyses demonstrate the value of combining multiple extraction and acquisition strategies to reveal chemical diversity beyond what metagenomics alone can capture.

Endometabolomic profiles mirrored *Microcystis* phylogeny, reflecting cyanobacterial dominance in biomass and metabolite production. Most annotated compounds belonged to small peptides, glycerolipids, and oligopeptides, which discriminated among phycospheres. The tight coupling between *Microcystis* genospecies and chemical profiles aligns with recent reports linking genotypes and chemotypes in environmental phycospheres [65]. Among the phycosphere-specific chemical signatures, we surveyed the oligopeptide and saccharide signals. Our results did not indicate that metabolome composition was directly related to the colonial morphology of the cultured strains, as we did not detect distinct saccharide profiles associated with differential mucilage production in strains that remained colonial under laboratory conditions. Cyanobacterial oligopeptides, known for their structural diversity and inhibitory activities, likely contribute to metabolic plasticity and defense [83–85]. Saccharide analyses revealed the importance of paulomycins, particularly in genospecies F, although their typical producer *Streptomyces* [86], was absent from HQ MAGs. This may reflect low-abundance or uncharacterized producers, or limitations of *in silico* metabolite annotation.

*Phyco1365* deviated from the others with distinct BGC content, taxonomic structure, phycosphere GSMNs, and metabolomic profiles. Differences in endometabolome content are likely due to low cyanobacterial abundance, distinct AB, and the near-exclusive presence of *Fimbriimonadia*, the latter being also observed at a lower abundance in *Phyco1364*, the second endometabolome outlier. These differences suggest altered microbial interactions but may also reflect the incompleteness of reference databases.

### Ecological insights from cultivated freshwater phycospheres

Recent studies have shown that metabolomic profiles of environmental *Microcystis* strains closely reflect their genotype, likely shaped by local environmental conditions [65]. Although our study relies on cultivated phycospheres, our results support this genotype–chemotype link and its ecological relevance. While Huré *et al*. [65] identified 13 chemotypes from 65 strains collected from 13 lakes, here we detected six distinct genotypes within a single site, indicating that multiple genotypes can coexist under similar environmental conditions and that AB patterns may persist under laboratory cultivation.

Several annotated compounds have reported allelopathic or bactericidal properties, including sorbistin A2, ornibactin C6/C8, and paulomycins [86–88], and may contribute to shaping phycosphere bacterial communities. While the annotations of sorbistin A2 and ornibactin C6/C8 are supported by the taxonomic composition of the phycosphere communities, all assignments are based exclusively on *in silico* predictions and should be interpreted with caution.

Regarding toxin production, the inability of some *Microcystis* strains to produce microcystins did not result in major differences in the overall phycosphere metabolism. How microcystin-producing and non-microcystin-producing strains interact at the bloom scale remains unresolved, but our modeling approach highlights functional complementarity, supporting potential *phycosphere*–*phycosphere* interactions at fine spatial scales [89]. These interpretations remain limited by incomplete reference databases, meaning GSMN reconstructions provide only a partial view of metabolism [67]. Given the number of analyzed genomes, we therefore relied on discrete modeling approaches [14, 47, 90]. Although cultivated phycospheres do not fully recapitulate bloom conditions, they nonetheless reveal substantial diversity and potential interaction networks, offering valuable insights into freshwater microbial ecology.

### Intra- and inter-phycosphere metabolic interactions: ecological perspectives from metabolic modeling

This study explored micro-diversity at multiple organizational levels. We first characterized intra-population genomic micro-diversity within the genus *Microcystis* across 12 naturally co-occurring isolates, consistent with patterns previously reported during bloom events [91, 92]. In parallel, micro-diversity was also observed within the AB communities of each phycosphere, with strong congruence between cyanobacterial genotypes and their bacterial partners. Although isolation and laboratory cultivation reduced community complexity, core *Microcystis*–AB interactions appeared to be preserved across genospecies. Together, these results extend the well-described micro-diversity of *Microcystis* to the phycosphere level, where it is thought to support adaptation to fine-scale ecological niches [91].

Metabolic complementarity simulations further indicated that phycosphere members could collectively expand their metabolic repertoire beyond individual organismal capacities. Rather than solely reflecting functional redundancy, this complementarity would allow access to otherwise unreachable metabolites and align with previous observations of cooperative metabolism in cyanobacteria-associated communities [17]. Maintaining phycosphere micro-diversity at small spatial scales could contribute to resource use efficiency by AB and support *Microcystis* growth, contributing to bloom persistence and providing a more relevant framework for modeling bloom dynamics [93].

When modeling interactions among the 12 phycospheres, an additional increase in metabolic potential emerged from simulation results, suggesting that phycosphere metabolism could be shaped not only by *Microcystis*–AB interactions but also by complementary processes operating across neighbouring communities. During bloom events, closely co-occurring phycospheres may thus be viewed as neighbour ecotypes represented by distinct genotypes. We therefore hypothesize that environmental adaptation during blooms could be explored at the metacommunity level, and that maintaining phycosphere micro-diversity through interactions among closely related phycospheres may represent a potential ecological advantage.

However, this inter-phycosphere framework is based solely on *in silico* predictions of metabolic potential and does not account for the spatial structure of natural blooms. *In situ*, colony morphology and mucilage may constrain metabolite diffusion, while mixing processes and free-living bacterioplankton could dilute or uncouple potential exchanges between phycospheres. Moreover, model predictions remain limited by database completeness and annotation accuracy. These hypotheses therefore require further investigation and experimental validation under spatially resolved and environmentally realistic conditions.

Overall, the systems biology framework adopted here demonstrates the value of integrating metagenomics, metabolomics, and metabolic modeling to investigate the structure and function of *Microcystis* phycospheres. We show that *Microcystis* phylogeny influences both bacterial recruitment and the chemical landscape, while simulations highlight a potential for metabolic cross-feeding both within and across phycospheres. These predictions provide a structured framework for hypothesis-driven investigations of mutualistic interactions and resource-sharing strategies in *in situ* metacommunities across contrasting ecotypes, which now require targeted experimental validation.

## Supplementary Material

Supplementary_material_ycag226

## Data Availability

Raw reads and assembled genomes are available on EBI ENA under accession number PRJEB102163. Metabolomic data is available on MetaboLights [50] with the study identifier MTBLS13909. Processed data related to the analyses are shared on Recherche DataGouv https://doi.org/10.57745/2P0IKJ. Scripts to perform data analysis are available on https://gitlab.inria.fr/comic-project/COMIC-Metabo.
