# Supplementary material for: Integrating metagenome-scale metabolic models and metabolomics to explore candidate biochemical interactions in cultivated Microcystis phycospheres
Source: ISME Commun. 2026 Aug 5;6(1):ycag226. doi: 10.1093/ismeco/ycag226 (PMC13533211; doi:10.1093/ismeco/ycag226)
Supplement: Supplementary file 1 [file supplementary_material_ycag226.zip › supplementary_material_ycag226.pdf]

Supplementary content for "Integrating metagenome-scale metabolic modelling and metabolomics to explore candidate biochemical interactions in cultivated *Microcystis* phycospheres"

Juliette Audemard[1,2], Nicolas Creusot[3], Julie Leloup[6], Charlotte Duval[4], Sébastien Halary[4], Lou Mary[4], Melissa Eon[3], Thomas Forjonel[3], Mohamed Mouffok[2], Rémy Puppo[4], Elodie Belmonte[8], Veronique Gautier[8], Jeanne Got[5], Marie Lefebvre[2], Gabriel V. Markov[7], Coralie Muller[1], Benjamin Marie[4], Binta Diémé[2*], Clémence Frioux[1*].

[1]Inria, University of Bordeaux, INRAE, F-33400 Talence, France

[2]Université Clermont Auvergne, INRAE, UNH, PFEM, MetaboHUB Clermont, 63000 Clermont-Ferrand, France

[3]INRAE, UR1454 EABX, Bordeaux Metabolome, MetaboHub Bordeaux, 33612 Gazinet Cestas, France

[4]Muséum National d'Histoire Naturelle, UMR 7245 CNRS-MNHN, MCAM, Paris, France

[5]Université de Rennes, Inria, CNRS, IRISA, Rennes, France

[6]Sorbonne Université, Univ Paris-Cité, Univ Paris-Est, CNRS, IRD, INRAE, IEES Paris, F-75005

[7]Sorbonne Université, CNRS, Laboratoire de Biologie Intégrative des Modèles Marins, LBI2M, F-29680 Roscoff, France

[8]Plateforme Gentyane, UMR 1095 Génétique Diversité Ecophysiologie des Céréales INRAE, UCA, Clermont-Ferrand, France

* Corresponding authors, equal contributions. binta.dieme@inrae.fr and clemence.frioux@inria.fr

1 Supplementary material and methods

1.1 Metagenomic sequencing

Single-molecule Real-time long reads sequencing was performed at Gentyane Sequencing Platform (Clermont-Ferrand, France) with a PacBio Revio Sequencer (Pacific Biosciences, Menlo Park, CA, USA). A library was prepared using the SMRTbell® Template Prep kit 3.0 and SMRTbell Barcoded adapter 3.0 (Pacific Biosciences, Menlo Park, CA, USA). For each sample, DNA was sheared using g-tubes (Covaris, England) generating DNA fragments of approximately 9 kb. Size distribution was determined using a fragment Analyzer (Agilent Technologies, Santa Clara, CA, USA). Around 300 ng of each DNA sample were treated to remove single-strand overhangs and repair DNA damage. Ligation with overhang adapters to both double-stranded DNA ends was performed to create a closed, single-stranded circular DNA. After a nuclease treatment, size-selection was done on pooled samples to remove fragments less than 5Kb, with 3.1X 35% diluted AMPure PacBio beads. Quality and quantify of the libraries were determined using a Fragment Analyzer (Agilent Technologies) and a Qubit fluorimeter (Life Technologies). A ready-to-sequence SMRTBell Polymerase Complex was created using a Revio polymerase kit (PacBio). The PacBio Revio instrument was programmed to load a 300 pM library and sequenced in CCS mode on a PacBio SMRTcell 25M, with the Revio Sequencing Plate (Pacific Biosciences) and 24-h acquiring movie.

1.2 Phylogenomic analysis of *Microcystis*

Normalised Robinson-Foulds metric [26] was used to assess the congruence between *Microcystis* phylogenetic tree and the dendrogram of its associated bacteria (AB). The phylogenetic tree was generated as described in the phylogenomic section of Methods (main manuscript). Associated bacteria presence/absence matrix was used to compute an AB jaccard distance matrix, using the vegdist

function from the R library vegan (v2.7-1), for each taxonomic level (*i.e.* species, genus, family, order, class, phylum). Hierarchical clustering was performed on the AB distance matrix using the hclust function (R stats library v4.5.0), with complete linkage method, generating a dendrogram of the distance between *Microcystis* strains relatively to their associated bacteria. Robinson-Foulds' metric was then computed to compare topologies of the host phylogenetic tree and the AB dendrogram, using the R package Treedist (v2.11.0). Representation of the conserved splits was realized using the dendlist and tanglegram functions from the R package dendextend (v1.19.1).

For tanglegram representations involving the comparison of phylogeny to other hierarchical relationships, we pruned the phylogenetic tree, re-rooted it to get genospecies C as the outgroup, and made it ultrametric using phytools (v2.3_0) non-negative least square method.

1.3 Functional annotation

We curated the identification of certain functions during metabolic network reconstruction of *Microcystis*. To handle potentially missing or mis-annotated functions, we systematically conducted a reciprocal-blast procedure. For the suspected false negatives, we blasted the entire MAGs on a local database of the researched functions [5]. We considered a protein sequence as homologous to one (preferably multiple) hits in the database if the following thresholds were respected: identity $\geq$ 80% and p-value $\leq$ 0.05. To double-check the protein characterisation, and avoid false positives, we then re-blasted it against the Uniprot database [8] and assessed whether another function was actually a better match. AntiSMASH outputs were a supplementary argument to support the identification of the putatively missing genes clusters.

1.4 Genome-scale metabolic network curation

Manual curation of GSMNs focused mainly on the metabolic networks of *Microcystis*, specifically the tricarboxylic acid cycle, and the microcystin-LR biosynthesis pathway. Automatic reconstruction of *Microcystis* GSMNs did not recover the key 2-oxoglutarate decarboxylase of the cyanobacterial tricarboxylic acid cycle [32]. Through the double-blast procedure described in Supplementary material and methods, we identified the gene associated to the latter enzyme (EC 4.1.1.71) in all *Microcystis* MAGs. The reaction catalysed by it, annotated under the ID *RXN-7774* in MetaCyc (v.27.0) [6] was then manually added in their GSMNs.
The same manual curation process was conducted for the microcystin-LR biosynthesis process when associated genes were identified. While D-methylaspartate is most likely the building block of MC-LR [21], no biosynthesis pathway has been described for it. However, synthesis of L-methylaspartate by cyanobacteria has been proposed [24, 21], which we integrated accordingly in *Microcystis* GSMNs. Although no subsequent isomerisation has been defined to go from L-methylaspartate to D-methylaspartate, such mechanism could putatively explain the latter form usage in MC-LR biosynthesis. The added reactions corresponds to MetaCyc entries *RXN-7743*, *RXN-7744*, *RXN-7751*, *METHYLOAA-OXYDOREDUCTASE-RXN*. Finally, we added a global reaction of MC-LR biosynthesis following literature knowledge on involved enzymes, described in Supp. Table **S8**.

1.5 Genome-scale metabolic network comparison

GSMNs comparison was performed based on their reaction contents using PADMet-analysis [1]. To compare *Microcystis* GSMN to its phylogeny, a tanglegram was constructed on a hierarchical clustering using complete linkage method and Jaccard distance of reactions presence-absence matrix. To compare GSMN contents at the phycosphere level, we performed a metric multidimensional scaling (metric MDS, based on a Bray-Curtis dissimilarity) taking into account AB relative abundances. First, we computed reactions presence-absence matrix of all members per phycosphere, then weighted those presence using the genome relative abundance in the community according to metagenomic data. Finally, we summed the weighted values to obtain a value for each reaction in each phycosphere.

1.6 Metabolic modelling

The modelling framework used by Metage2Metabo [3] relies on network expansion [11]. Briefly, each metabolite of the GSMN is either reached (producible) in the simulated conditions, or non-reached (unproducible). Network expansion states that products of a reaction are reached only if all of the reaction's reactants are reached or belong to the seeds. M2M distinguishes the metabolites that can

be reached individually by GSMNs from those relying on metabolic complementarity and mutualistic interactions to be producible.

Metabolic modelling simulations require a list of compounds called *seeds* that initiate the reachability of molecules in the metabolic networks. Following the composition of BG11 growth medium used experimentally, Supp. Table **S1** describes the seeds used in the models.

Two types of simulations were performed. The first one is at the phycosphere level, simulating lab culture conditions of each community and estimating the *intra-phycosphere metabolic complementarity*. For each community, the respective GSMNs of the cyanobacterium and of its associated bacteria were gathered and M2M was run with the command `metacom`. The second simulation aimed at modelling the interactions among distinct phycospheres in lake-like natural conditions. For this, each collection of GSMNs related to a phycosphere was merged with the PADMet library, including the pseudogenomes GSMNs. The simulated community consisted of twelve phycosphere-level networks, enabling to measure the *inter-phycosphere metabolic complementarity* potential. Resulting metabolic potentials (reachable metabolites by the whole community or by individual community members) were then analysed. Producible compounds were grouped using the metabolite classification of natural products from [18].

Name	Chemical Formula	MetaCyc ID
adp*	C10H12N5O10P2	ADP
ammonium	NH4+	AMMONIUM
biotin	C10H15N2O3S	BIOTIN
boric acid	H3O3B	BORATE
calcium	Ca+	CA+2
calcium chloride dihydrate	CaCl2	CPD0-2507
carbon dioxide	CO2	CARBON-DIOXIDE
chloride	Cl	CL-
citrate	C6H5O7	CIT
cobalt(II)	Co+2	CO+2
cobalt(II) nitrate	Co(NO3)2	CPD-22943
copper(II)	Cu+2	CU+2
copper(II) sulfate	Cu(SO4)	CUSO4
coA*	C21H32N7O16P3S	CO-A
cyanocob(III)alamin	C63H88N14O14PCo	CPD-315
dioxygen	O2	OXYGEN-MOLECULE
dipotassium phosphate	K2HPO4	CPD0-2433
EDTA disodium salt	Na2(C10N2H14O8)	CPD-19640
ferric ammonium citrate	Fe(NH4)(C6H5O7)	CPD-19639
folate class*		THF-GLU-N
hepes solution	C8H18N2O4S	HEPES
hydrogen chloride	HCl	HCL
iron(III)	Fe+3	FE+3
light		Light
manganese(II)	Mn+2	MN+2
manganese(II) sulfate	Mn(SO4)	CPD0-1608
molybdate	O4Mo	CPD-3
nad*	C21H26N7O14P2	NAD
nadp*	C21H25N7O17P3	NADP
NAD ou NADP*		NAD-P-OR-NOP
nitrate	NO3	NITRATE
phosphate	PO4	Pi
potassium	K+	K+
sodium	Na+	NA+
sodium molybdate dihydrate	Na2(MoO4)(H2O)2	CPD-19634
sodium nitrate	Na(NO3)	CPD-12921
sulfate	SO4-2	SULFATE
sulfide	H2S	HS
thiamine	C12H17N4OS	THIAMINE
thiamine HCl	(HCl)(C12N4H17SO)	CPD-22876
ubiquinones*		Ubiquinones
water	H2O	WATER
zinc	Zn+2	ZN+2
zinc sulfate	Zn(SO4)	CPD0-2393

Supplementary Table S1: **Adapted seed list from the BG11 culture medium**. An asterisk (∗) follows cofactors that are not part of BG11's composition, but are added *in silico* to initiate reachability, as they are common and important enough for basic metabolic process to be included in the seeds.

1.7 Metabolomic data acquisition and analyses

1.7.1 Monophasic (MP) and biphasic (BP) metabolomic extractions protocols

In the MP method, about 1 mg of lyophilised biomass or supernatant was extracted with 100 µL of solvent mix of methanol/water (3:1, v/v) acidified with 0.1 percent formic acid. Metabolite extraction was carried out on ice using an ultrasonic probe, performing four consecutive cycles of 30 seconds, each followed by a 30-second pause. The samples were then centrifuged (15,000 *g*, 4°C, 10 minutes). A volume of 50 µL of the supernatant was transferred into injection vials, and 1 µL was analysed by ultra-high-performance liquid chromatography (Elute, Bruker, Bremen, Germany) coupled with time of flight high-resolution tandem mass spectrometry (Maxis II-QTOF, Bruker, Bremen, Germany) (UHPLC-HRMS/MS).

In the BP method, about 2 mg of dried biomass was first extracted on ice by adding 1 mL of cold

mixture of methyl-tert-butyl-ether and methanol (MTBE-MeOH, ratio 3:1, v:v) and homogenisation during 2 cycle of 15-second (6000 RPM, FastPrep-24 5G, MP-biomededical). Then, a 650 µL cold mixture of ultrapure water and methanol (UPW-MeOH, ratio 3:1, v:v) was added, followed by a third cycle of homogenization. Hydrophilic and lipophylic fractions were separated by centrifugation (12000 RPM, 4°C, 5 min) and further partly collected. A second similar extraction was performed to optimise metabolite recovery after the addition of 700 µL of MTBE-MeOH mixture and 455 μL of UPW-MeOH mixture. In total, lipophilic and hydrophilic fractions consisted in 1.1 mL and 1.3 mL extracts, respectively. Following the extraction, 500 µL of each fractions were evaported to dryness (EZ2-PLUS, Genevac) and resuspended in ACN:H20 (1:1, v/v) mixture and ISO:ACN (1:1, v/v) respectively for hydrophilic and lipophilic fractions. Both fractions were analysed on UHPLC Vanquish combined to Q-Exactive Plus (Qex+) HRMS equiped with a heated electropray ionisation probe (HESI-II) (ThermoFisher Scientific, San Jose, CA).

1.7.2 Elution gradients

MP extract was separated on a C18 Acclaim PolarAdvantage II column (2.1x100 mm, 2.2 µm, ThermoScientific), as described in Supp. Table **S2**.

Time (min)	Flow (mL min^{-1})	Eluent A (%)	Eluent B (%)
0	0.300	95	5
2	0.3	95	5
16	0.300	10	90
18	0.300	10	90
19	0.300	95	5
21	0.300	95	5

Supplementary Table S2: **Gradient used to elute the monophasic extracts on C18 Acclaim PolarAdvantage II column at 40°C**. Eluent A, UPW supplemented with 0.08% of FA; Eluent B, ACN supplemented with 0.08% of FA.

BP extracts were separated on HSST3 RP-C18 column (150 × 2.1 mm × 1.7 µm, Waters) to analyse hydrophilic and lipophilic fractions (Supp. Tables **S3**, **S4**)) and BEH-Amide (100 x 2.1 mm x 1.7 µm, Waters) to analyse only hydrophilic fraction (Supp. Table **S5**).

Time (min)	Flow (mL min^{-1})	Eluent A (%)	Eluent B (%)
0	0.400	99	1
4	0.400	99	1
20	0.400	1	99
22	0.400	1	99
22.1	0.400	99	1
25	0.400	99	1

Supplementary Table S3: **Gradient used to elute the hydrophilic fraction on HSST3 column at 45°C**. Eluent A, UPW supplemented with 0.1 % of FA ; Eluent B, ACN supplemented with 0.1 % of FA.

Time (min)	Flow (mL min-1)	Eluent A (%)	Eluent B (%)
0	0.300	70	30
4	0.300	55	45
22	0.300	30	70
24	0.300	1	99
26	0.300	1	99
30	0.300	70	30

Supplementary Table S4: **Gradient used to elute the lipophilic fraction on HSST3 column at 45°C**. Eluent A, UPW/ACN (60:40, v:v) supplemented with 0.1 % of FA ; Eluent B, ACN/isopropanol (10:90, v:v) supplemented with 0.1 % of FA.

Time (min)	Flow (mL min^{-1})	Eluent A (%)	Eluent B (%)
0	0.35	20	80
2	0.35	20	80
12	0.35	80	20
16	0.35	80	20
18	0.35	20	80
24	0.35	20	80

Supplementary Table S5: **Gradient used to elute the hydrophilic fraction on BEH-Amide**. Eluent A, UPW supplemented with ammonium acetate 0.8% / EtOH (95:5; v:v) ; Eluent B, ACN supplement with ammonium acetate 0.8% / EtOH (95:5; v:v).

1.7.3 Data acquisition

For MP, each extract was analysed in triplicate in positive ionization mode with a mass range of 50–1500 *m/z*, and automatic fragmentation of the TOP 5 most intense ions (autoMS/MS). QC and Blank samples (injected every six samples and every triplicate, respectively) were examined to ensure the reproducibility and robustness of the whole data series.

For BP, samples were also analyses in triplicates on QEx+ in both positive and negative mode according the parameters presented in Supp. Table **S6**.

	DDA full scan (top 5)	**DDA MS/MS**
**Scan Range (m/z)**	50-1500	
**Resolution (FWHM)**	70,000	17,500
**Automated Gain Control (AGC)**	3e6	1e5
**Spray Voltage (kV)**	Pos : +4 ; Neg : -3.5	4
**Sheath Gas Flow rate (µA)**	Pos : 40 ; Neg : 30	40
**Capillary Temperature (°C)**	Pos : 350 ; Neg : 300	350
**NCE**	-	50
**Loop Count**	-	2

Supplementary Table S6: **Qex+ parameters for the analysis of BP extracts** after hydrophilic, lipophilic and HILIC separation

.

1.7.4 Data processing, annotation and chemometrics

In order to highlight specialised metabolites, MetaboScape software(R) (Bruker Daltonics, Bremen, Germany) was first used to process only MP data. After recalibration of each sample analysis (< 1ppm, according to sodium formate internal standard), peaks with intensities greater than 5,000 counts in at least 10% of the set of samples and minimal RT-correlation coefficient of 0.7. Different states of charge (1+, 2+ and 3+) and classical adducts were grouped together, and the peak area was determined in order to generate a unique global data matrix containing semi-quantification results for each metabolite in all analysed samples. Feature annotations were attempted according to respective ion mass (< 2ppm) and isotopic pattern (< 20msigma) by automatic match with the CyanometDB 1.0 database [16] which contains over 2,100 chemical formulas of known specialised metabolite produced by cyanobacteria.

For untargeted metabolomics processing, MSDial software was used to extract features from samples. MSDial parameters for both MP and BP extracts are provided in Supp. Table **S7**.

	QTOF (Monophasic)	Qexactive+_ Orbitrap (Biphasic)
MSDIAL version	v5.5.251021	v5.5.251021
**Conversion**		
Software or package (specify language)	no	ThermoParser
Spectrum	-	mzML
MS levels	-	All
Peak Picking	-	All
Metadata	-	TXT
All detectors	-	Yes
Exceptions	-	Exclude reference and exception data
Gzipped	-	No
Compression	-	None
Errors	-	Ignore missing instrument properties
**Data Collection**		
Mass accuracy		
MS1 tolerance	0.01	0.01
MS2 tolerance	0.05	0.05
Advanced - Data collection		
Rt time begin	0	0
Rt time range end	100	100
MS1 mass range begin	0	0
MS1 mass range end	2000	2000
MS/MS mass range begin	0	0
MS/MS mass range end	2000	2000
Excute Rt correction	NO	NO
Advanced - Isotope recognition		
maximum number of isotopes	5	5
maximum number of charges	1	1
Consider Cl and Br Elements	YES	YES
Multithreading		
Number of threads	40	40
**Peak Detection**		
Peak detection parameters		
Minimum peak height	2.E+03	5.E+05
Mass slice width	0.1	0.1
Advanced		
Smoothing method	Linear weighet moving average	Linear weighet moving average
Smoothing level	3	3
Minimum peak width	5	5
Exclusion mass list	NO	NO
**Spectrum deconvolution**		
Deconvolution parameters		
Sigma window value	0.5	0.5
MS/MS abundance cut off	25	10
Advanced		
Exlucde after precursor ion	YES	YES
Keep the isotopic ion until	5	5
Kepp the isotopic ions w/o MS2Dec	YES	YES
**Identification**		
Database setting		
Name	FragHub	FragHub
Last updated	24-Apr-24	24-Apr-24
Downloaded from	\href{https://zenodo.org/records/11057687}{https://zenodo.org/records/11057687 }	\href{https://zenodo.org/records/11057687}{https://zenodo.org/records/11057687 }
Comments	MS/MS positive mode	MS/MS positive mode
Comments	Combination of currated GNPS, Mslib, MassBank, Riken, Mona	Combination of currated GNPS, MsLib, MassBank, Riken, Mona
Annotation Method		
Accurate Mass Tolerance (MS1)	0.015 Da	0.01 Da
Accurate Mass Tolerance (MS2)	0.03 Da	0.05 Da
Retention time Tolerance	100	100
MS2 spectrum cut off		
spectrum amplitude cut off (relative)	0	0
spectrum amplitude cut off (absolute)	0	0
Mass range begin (Da)	0	0
Mass range begin (Da)	2000	2000
Annotation cut off		
Dot product score	600	600
weighted dot product score	600	600
reverse dot product score	800	800
Matched spectrum percentage (%)	50%	25%
Minimum number of matched spectrum	5	3
Retention time scoring		
Use retention time or scoring	no	no
use retention time for filtering	no	no
Adduct ion		
Data setting	[M+H]+, [M+NH4]+, [M+Na]+, [M+CH3OH+H]+, [M+K]+, [M+Li]+, [M+ACN+H]+, [M+H-H2O]+, [M+H-2H2O]+, [M+2Na-H]+, [M+ACN+Na]+, [M+2K-H]+, [M+2ACN+H]+, [M-C6H8O6+H]+, [2M+H]+, [2M+NH4]+, [2M+Na]+, [2M+3H2O+2H]+, [2M+K]+, [2M+ACN+H]+, [2M+ACN+Na]+, [M-H]-, [M-H2O-H]-, [M+Na-2H]-, [M+FA-H]-, [M-C6H10O4-H]-, [M-C6H10O5-H]-, [M-C6H8O6-H]-, [M+CH3COONa-H]-, [2M-H]-, [2M+FA-H]-.	[M+H]+, [M+NH4]+, [M+Na]+, [M+CH3OH+H]+, [M+K]+, [M+Li]+, [M+ACN+H]+, [M+H-H2O]+, [M+H-2H2O]+, [M+2Na-H]+, [M+ACN+Na]+, [M+2K-H]+, [M+2ACN+H]+, [M-C6H8O6+H]+, [2M+H]+, [2M+NH4]+, [2M+Na]+, [2M+3H2O+2H]+, [2M+K]+, [2M+ACN+H]+, [2M+ACN+Na]+, M-H]-, [M-H2O-H]-, [M+Na-2H]-, [M+FA-H]-, [M-C6H10O4-H]-, [M-C6H10O5-H]-, [M-C6H8O6-H]-, [M+CH3COONa-H]-, [2M-H]-, [2M+FA-H]-.
**Alignment parameters**		
Alignment parameters setting		
Result Name	x	x
Reference File	First QC	First QC
Retention time tolerance	0.2	0.2
MS1 tolerance	0.025	0.025
Advanced		
Retention time factor	0.5	0.5
MS1 factor	0.5	0.5
Peak count filter	0	0
N% detected in at least one group	0	0
remove features based on blank information	NO	NO
Sample max / blank average	5	5
Keep reference match metabolites features	YES	YES
Keep suggested (w/o MS2) metabolites features	YES	YES
Keop removable features and assigne the tag	YES	YES
gap filling by compulsion	YES	NO
MScleanR		
Blank ratio	0.8	0.8
Delete Ghost Peak	YES	YES
Incorrect Mass	YES	YES
Relative Standard Deviation (max RSD)	50	50
Relative Mass Defect	50-3500	50-3500

Supplementary Table S7: **List of parameters in MS-DIAL** (v5.25) for processing metabolomics raw data and further filtering

Feature intensity was further normalised by the Total Ion Chromatogram (TIC) prior the use of MS-cleanR [12] to filter the data according blank intensity, RSD, RMD, etc. (parameters in Supp. Table **S7**.) Filtered data were finally export as peak lists (i.e. one per acquisition mode) for chemometrics analysis and as spectra (as individual .mat files) to implement SIRIUS (v6.1) [9]. SIRIUS software

allowed to interrogate structural libraries (SIRIUS database and cyanoMetDB [17]) and provide scoring of the formula (ZODIAC) and structure (CSI:FingerID). [10, 19, 29]. In has to be noted, that these filtered peak lists contained both "only MS1" features (i.e. detected signals for which no fragmentation was performed) and "MS2" features (i.e. signals for which a fragmentation was performed allowing to interrogate spectral or structural DBs).

Output files from MS-DIAL and SIRIUS (Top 50 per feature) were further imported into MS-Net (developed in Knime Analytics Platform, v5.2) [13] in order to merge Pos and Neg acquisition for each gradient (hydrophilic-HSST3-pos/neg, hydrophilic-HILIC-pos/neg, lipophilic-pos/neg), suppress analytical redundancy, provide annotation confidence level and classification (NPclassifier, [18]), and highlight link between metabolites and identified bacterial genus and *Microcystis* sp. Briefly, among the top 50 in silico candidates per feature, those matching taxonomic criteria (genus: *Gemmatimonas, Hydrogenophaga, Meiothermus, Neoroseomonas, Aminobacter, Limnobacter, Novosphingobium, Phenylobacterium, Nevskia, Rhabdaerophilum, Cnuella, Flavobacterium, Aquidulcibacter, Brevundimonas, Caulobacter, Silanimonas, Inhella, IdeonellaA, Pararheinheimera, Falsiroseomonas, PseudomonasE, Rubrivivax, LeptospiraA, Microcystis* ; Famillies : *Fimbriimonadaceae, Sphingomonadaceae, Gemmatimonadaceae, TH1-2, Burkholderiaceae, Thermaceae, Acetobacteraceae, Caulobacteraceae, Rhizobiaceae, Nevskiaceae, Beijerinckiaceae, Chitinophagaceae, Flavobacteriaceae, Tepidisphaeraceae, Xanthomonadaceae, Alteromonadaceae, Pseudomonadaceae, Usitatibacteraceae, Leptospiraceae, Microcystaceae*) were elevated to Level 3a.

Features were filtered using Ion Identity Network (IIN) results to remove redundant adducts and isotopes, followed by MS-CleanR-based RT clustering (ΔRT $\leq$ 0.01 min). Within each cluster, the top 2 features by network degree and the top 2 by peak intensity were retained. The MS2 network was constrained to edges with cosine similarity $\geq$ 0.7 and ΔRT $\leq$ 8 min between connected nodes. High-confidence annotations (Levels 1, 2a, 3a) seeded the MS2 network for iterative annotation propagation. For each feature pair, candidate structures were ranked using a weighting parameter set to $\alpha = 0.3$, prioritizing structural-spectral evidence (70%) over *in silico* ranking (30%). The top 5 candidates by Link Score were retained per feature before looping through the entire MS2 network. Redundant annotations with identical InChIKey identifiers and Pearson correlation among samples > 0.7g were consolidated by selecting the candidate with the highest mean peak height. Positive and negative mode feature lists were merged using ΔRT $\leq$ 0.05 min, Δm/z $\leq$ 0.005 Da, and a minimum Pearson correlation $\geq$ 0.6 across sample intensities. Final annotations were enriched with chemical ontology classifications from ClassyFire (kingdom, superclass, class, subclass) and NPClassifier (pathway, superclass, class). Database identifiers (PubChem CID, KEGG, HMDB, ChEBI) were retrieved using the Chemical Translation Service. Natural product-likeness scores were calculated using the NPlikeness calculator [15]. Structural similarity networks were constructed using Tanimoto coefficient $\geq$ 0.8, retaining the top 2 nearest neighbours per feature. The complete list of annotated features was provide in the file *Supplementary_tables.xlsx*

Regarding chemometrics, a principal component analysis (PCA) was first performed on each dataset to assess the consistency of the three technical replicates. These replicates were further averaged to keep one signal per metabolite for each phycosphere. Then, all the peak lists (MP, BP-hydro-HSST3-pos/neg, BP-hydro-HILIC-pos/neg, BP-lipo-pos/neg) were merged and filtered in order to remove redundancy by keeping single annotation based on InChIKey, while features with no MS2 might present redundancy (i.e. adduct from the same molecules). Feature intensity was normalised, transformed and scaled (*i.e.* Sum normalisation, cube root transformation and pareto scaling) before statistical analyses.

1.8 Comparison of GSMN contents and metabolic models with metabolomics

Mapping of the entire metabolomic dataset to GSMN contents (including the PGs) was performed with MetaNetMap (v1.0.1) [23] using MetaCyc (v.29.0) as a reference mapping database. Metabolite presence-absence data per phycosphere was obtained by intersecting metabolomic signal (requiring detection in $\geq$ 2 out of 3 replicates per community) with GSMN mapping. Network hit proportions were calculated as the number of networks mapped per metabolite divided by the number of members in the phycosphere. A heatmap was generated with communities as rows and metabolites as columns, stratified by NP-Classifier classes.

In addition, Venn diagram was generated using Jvenn [2] in order to compare annotated metabolites from metabolomics and predicted metabolites from GSMNs based on InChiKey.

Finally, the chemical landscape of *Phyco1364* was summarised as sunplot according the number of

features per NPC_pathway/superclasses/classes and further compared to this predicted from GSMNs modelling.

2 Supplementary results

2.1 Phylogenomic tree of *Microcystis*

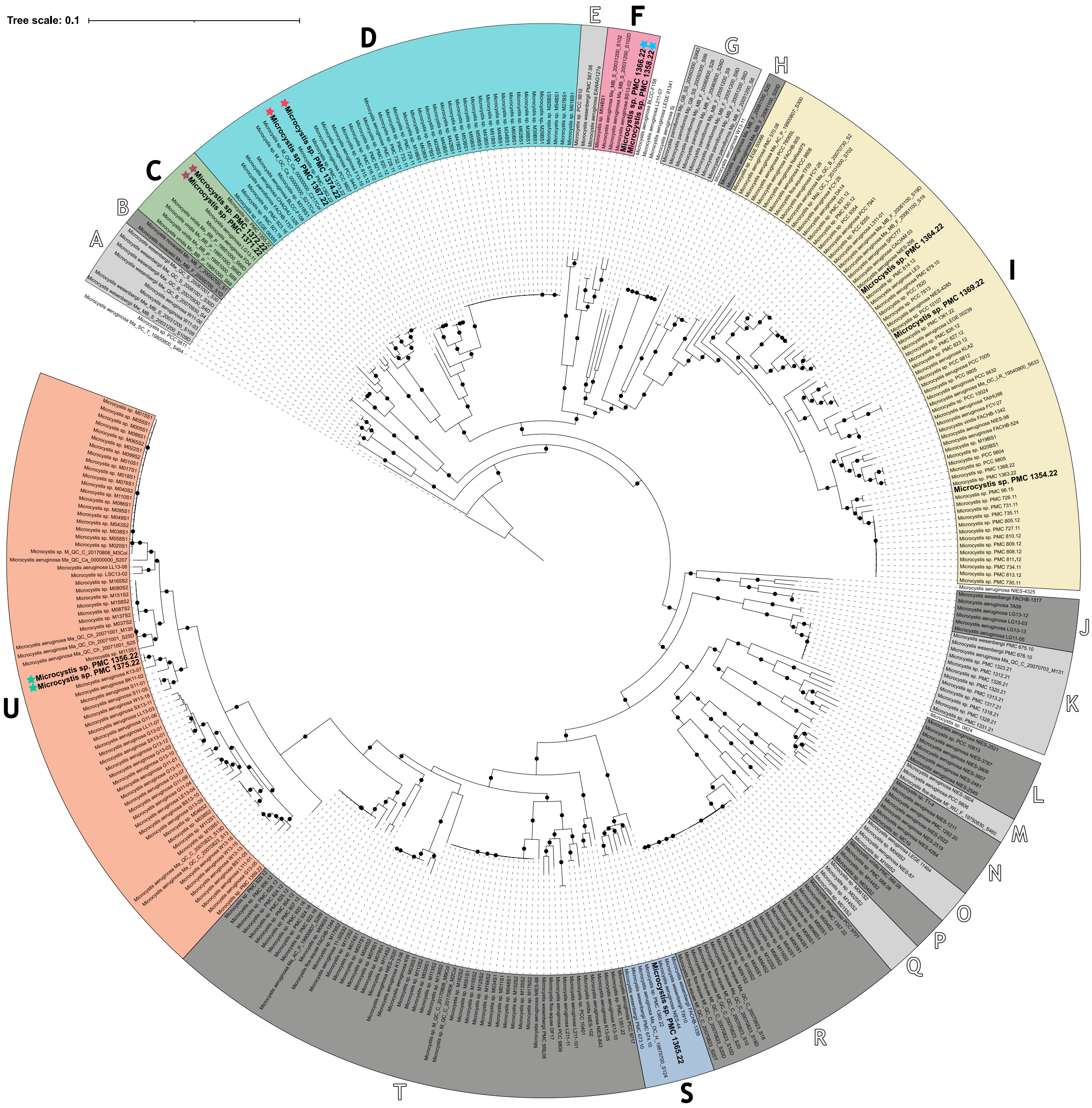

Supplementary Figure S1: **Maximum likelihood phylogenomic tree of *Microcystis* species.** The boxes group together strains from the same genospecies, labelled from A to U. The coloured ones (C, D, F, I, S and U) correspond to the genospecies of the 12 surveyed strains (in bold). The stars associated with the labels indicate that the associated strains belong to the same genotype. Tree branches supported by bootstrap values over 60% are indicated by a black circle. The tree is rooted on *M. aeruginosa* Ma_SC_T_19800800_S464, as determined in previous studies [4].

2.2 Taxonomy of *Microcystis*' associated microbiome

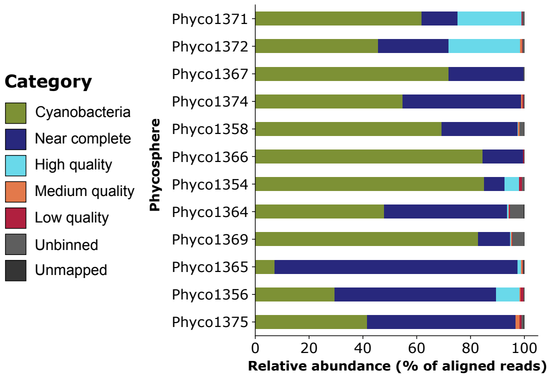

Supplementary Figure S2: **Relative abundance of Cyanobacteria, ABs (near complete, high, medium, or low quality MAGs), or unbinned contigs** according to metagenomic read mapping.

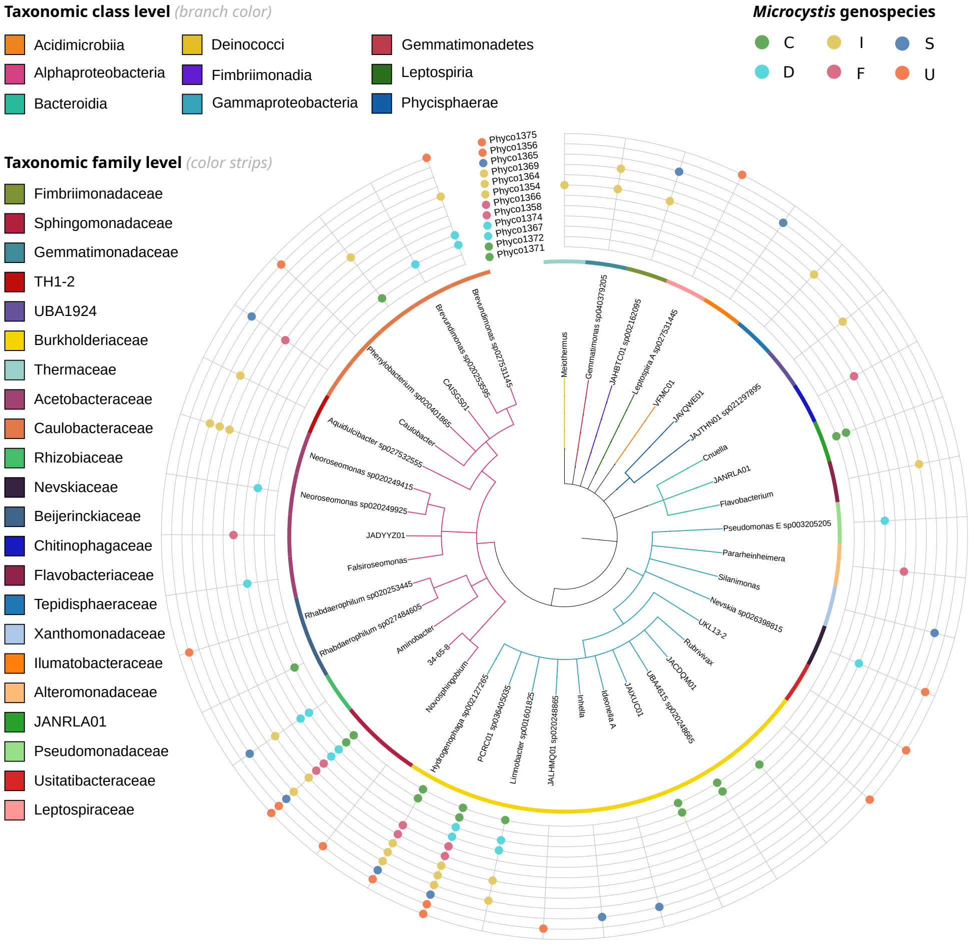

Supplementary Figure S3: **Phycospheres bacteria composition.** Taxonomic tree covering all distinct taxa identified from phycospheres high quality MAGs. The tree outer coloured circle annotates the Family of the bacteria, the branches are coloured by taxonomic Classes. On the very outer part, the grid identifies which taxa are found in which phycosphere; a coloured marble corresponding to the phycosphere is present when the taxa was annotated at least once in it.

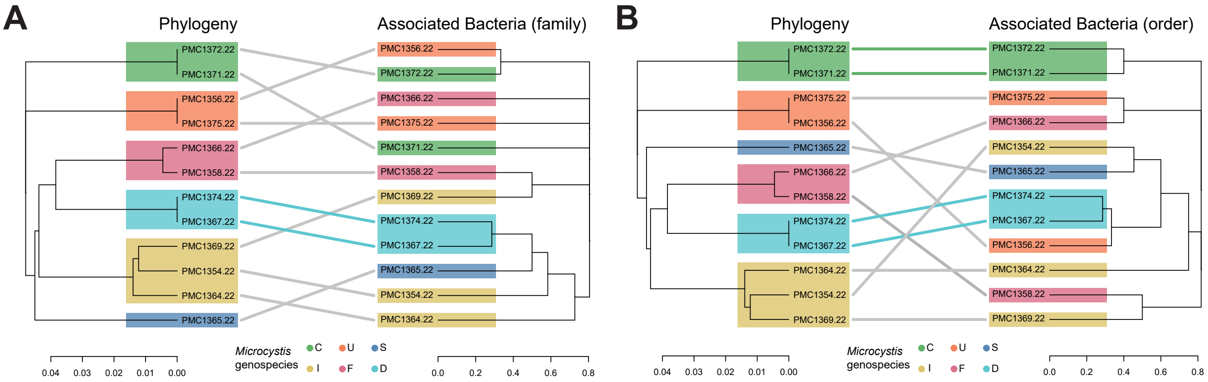

Supplementary Figure S4: **Relationships between *Microcystis* strain phylogenomy and the taxonomic composition of the respective associated bacteria according to hierarchical classification A.** Family taxonomic level. **B.** Order taxonomic level.

2.3 Microcystin-LR biosynthesis pathway

Enzyme	Type	Main function	References	Number of *Microcystis* with this gene
McyG	PKS-NRPS	Activation of initiator phenylpropanoid; elongation by Malonyl-CoA	[25, 14, 30]	5
McyJ	O-MT	O-methylation	[7, 30]	5
McyD	PKS	Elongation by Malonyl-CoA; Dehydration	[30]	5
McyE	PKS-NRPS	Elongation by D-Glu	[30]	5
McyF	Glutamate Racemase	Epimerization of L-Glu into D-Glu	[30, 27]	7
McyA	NRPS	Elongation by L-Ser and D-Ala; Epimerization of L-Ala into D-Ala	[30]	5
McyI	2-HADH	Production of D-MeAsp	[22, 24]	5
McyB	NRPS	Elongation by L-Leu and D-MeAsp	[30]	5
McyC	NRPS	Elongation by L-Arg; Cyclization	[30]	5
McyH	ABC transporter	Extracellular transport of MC-LR	[30]	5

Supplementary Table S8: **Description of the enzymes involved in the biosynthesis of microcystin-LR**, in their order of action. **Abbreviations: NRPS**, Non-Ribosomal Peptide Synthetase; **PKS**, Polyketide Synthase; **ABC transporter**, ATP-Binding Cassette transporter; **HADH**, Hydroxy Acid Dehydrogenase; **MT**, O-methyltransferase.

2.4 Phycosphere metabolic diversity

2.4.1 Metabolic annotations of associated bacteria (AB)

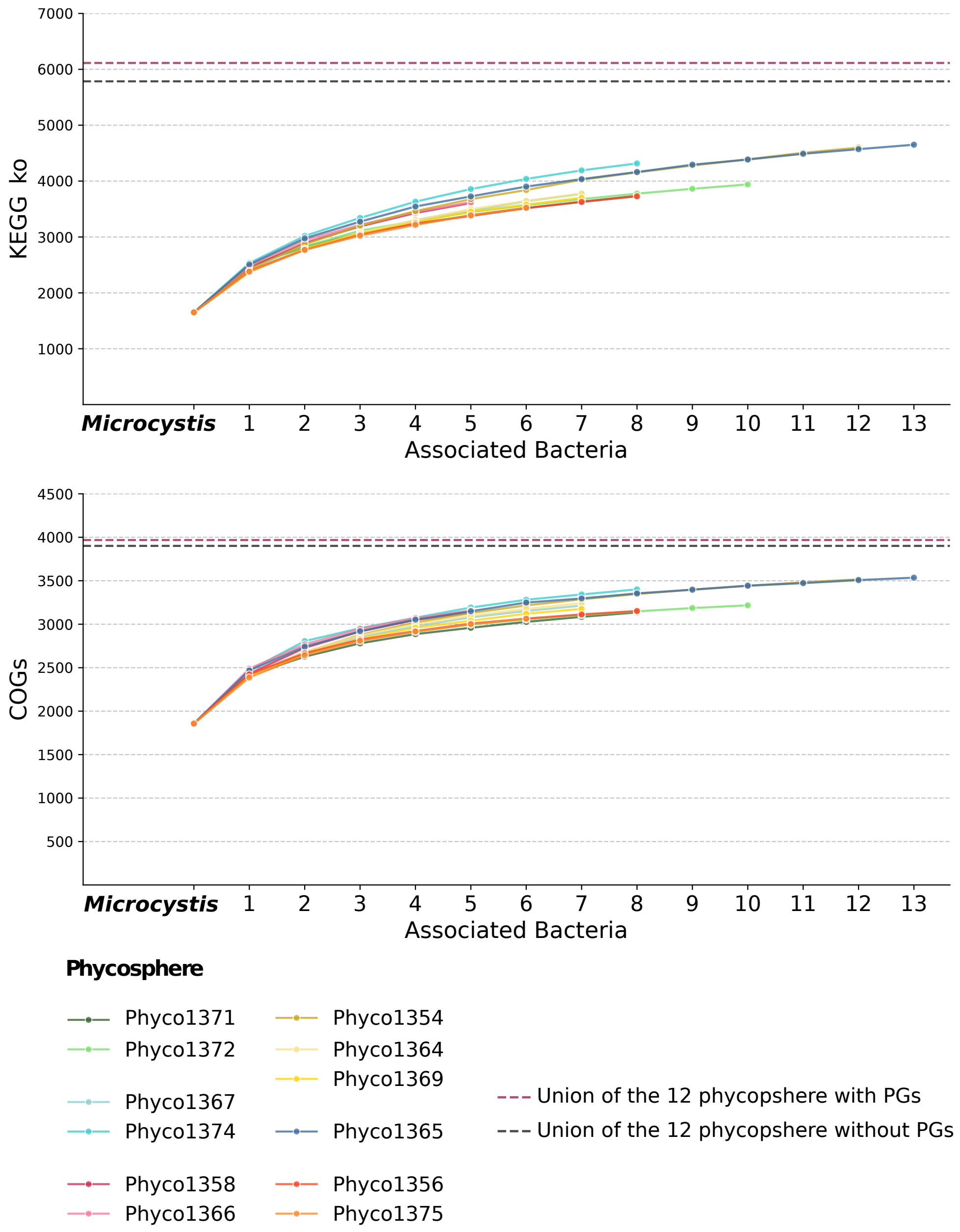

Supplementary Figure S5: **Rarefaction-curve of KEGG KO (above) and COGs (below) annotations** from phycosphere metagenomes. Rarefaction curves represent accumulation of unique annotations as a function of the number of sampled associated bacteria, based on 100 sampling iterations. The starting point is *Microcystis*. Pseudogenomes (PGs) are excluded from the sampling of phycosphere's individual curves.

2.4.2 Biogechemical cycles

Nitrogen Cycle For almost all phycospheres, at least one associated bacterium—and not *Microcystis*—carried the nitrate reductase genes *narGH* (EC:1.7.5.1), as well as nitrite reductase genes *NirBD* (EC:1.7.1.15) in every phycosphere. However, the nitrite reduction gene *NirA* (EC:1.7.7.1) was found in all *Microcystis* genomes. The NO-forming nitrite reductase gene *nirK* was detected in members of *Phyco1354*, *Phyco1365*, *Phyco1367*, *Phyco1374*, and *Phyco1375*, but not in *Microcystis*. Notably, these same phycosphere-associated bacteria were also the only ones to possess nitrite oxide

reductase genes *NorBD* (EC:1.7.2.5). No genes for nitrogen fixation were identified in any MAGs, either from *Microcystis* or the heterotrophic bacteria.

Methanogenesis We did not detect *McrABC* genes for methanogenesis in *Microcystis* genomes, although *Microcystis* are suspected to produce methane [33]. However, a gene for methane oxidation—the methane monooxygenase regulatory protein B—was found in non-cyanobacterial members in nine out of twelve phycospheres.

Complex Carbon As the bacteria associated with *Microcystis* in phycospheres are heterotrophic, they are expected to carry genes for the degradation of complex carbohydrates such as oligosaccharides. Indeed, in some phycospheres, genes for xylose, mannose, and galactose degradation were identified. Additionally, genes implicated in the breakdown of biomolecules originating from phytoplankton and fungi—including cellulose, hemicellulose, and pullulan—were also detected.

3 Metabolomic analyses

3.1 Added value of combined extraction protocols

Figure **S6** depicts the annotated (InChiKey identifiers) metabolomic signals obtained with each extraction protocol, highlighting their complementarity to provide a comprehensive picture of the phycosphere endometabolome.

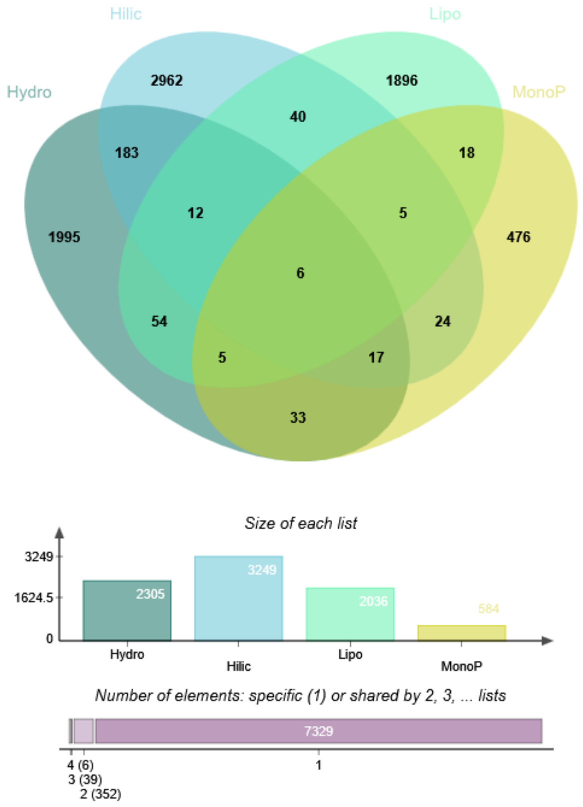

Supplementary Figure S6: **Venn diagram of putatively annotated features according to extraction protocols and chromatographic method**. The Venn diagram was generated with *jvenn* (`https://jvenn.toulouse.inra.fr/app/index.html`. **Abbreviations:** Hydro, Hydrophilic fraction separated on HSST3 Column; Hilic, Hydrophilic fraction separated on BEH-Amide column; Lipo, Lipophilic fraction separated on HSST3; MonoP, MP extract separated on C18 polar Advances II column.

3.2 Biochemical composition of the phycospheres

The principal components analysis (PCA) in Supp. Fig. **S7** shows a separation of the phycosphere regarding their endometabolome composition only partly reflecting *Microcystis* genotype, as noted in the HCA (Main Figure 4A of the manuscript).

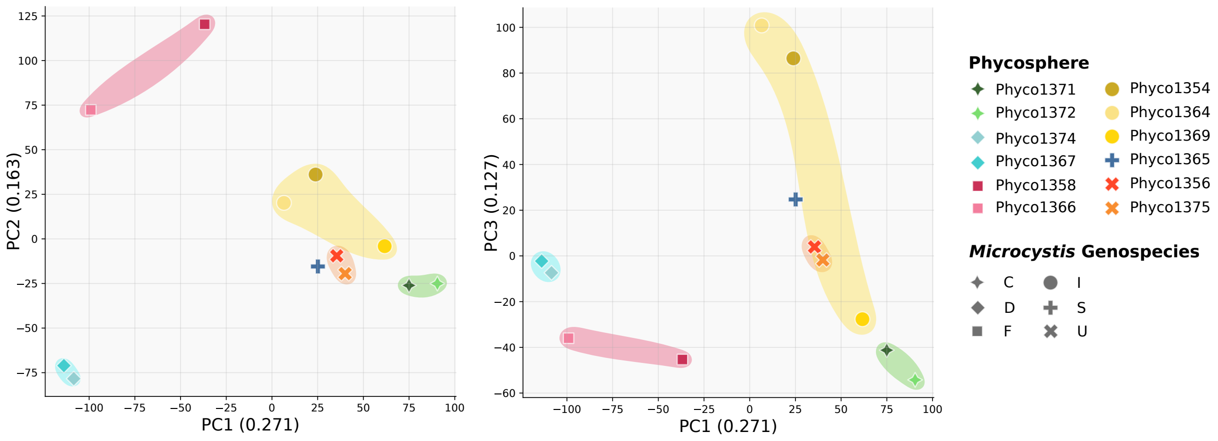

Supplementary Figure S7: **PCA of the transformed metabolomics signals.** For each of the extraction methods, triplicates were merged to get one signal per metabolite, and all extractions results aggregated. Coloured areas highlight phycosphere's associated to the same *Microcystis* genospecies.

Distribution of chemical classes (NPclassifier, [18]) relative intensity among the physcopheres are reported in Fig. **S8**. At the NPC-pathway level, the annotations do not showed marked differences in the biochemical composition of the twelve phycospheres.

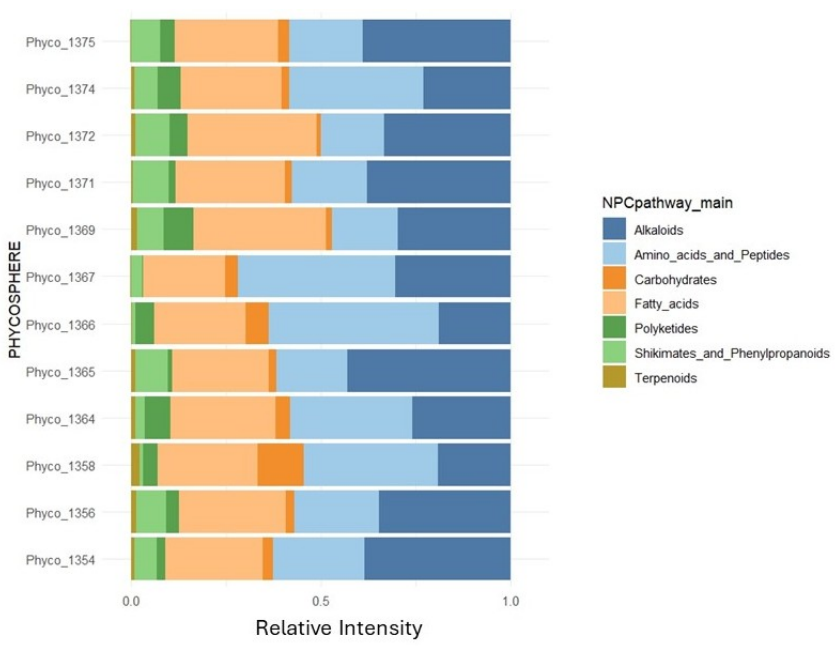

Supplementary Figure S8: **Barplot depicting the relative intensity of NPC Pathways among the phycospheres**.

3.3 Taxonomic assignments of metabolomic signals

MS-Net highlights that among the 6016 putatively annotated and classified metabolites, 144 were reported in some taxa composing the phycosphere from metagenomics (Fig. **S9**). The main represented genus and familly was Microcystis and Microcystae (63 features), likely in accordance with the higher abundance of this taxa in most of the Phycosphere and also the higher number of studies on

Microcystins specialised metabolites in the literature, on which COCONUT database is based. Then, 40 features were reported with *Pseudomonas* or *Pseudomonoceae* and 10 with *Burkholderiaceae*. Among them, two apocarotenoids (C30) were putatively annotated. These compounds are known to have antioxidant properties [28]. We also annotated compounds with bactericidal effects like Sorbistin A2 [31] or siderophores like Ornibactin C6 and C8 [20]. These compounds may offer an ecological advantage in productive phycospheres. However, as they are annotated *in silico*, these results should be treated with caution.

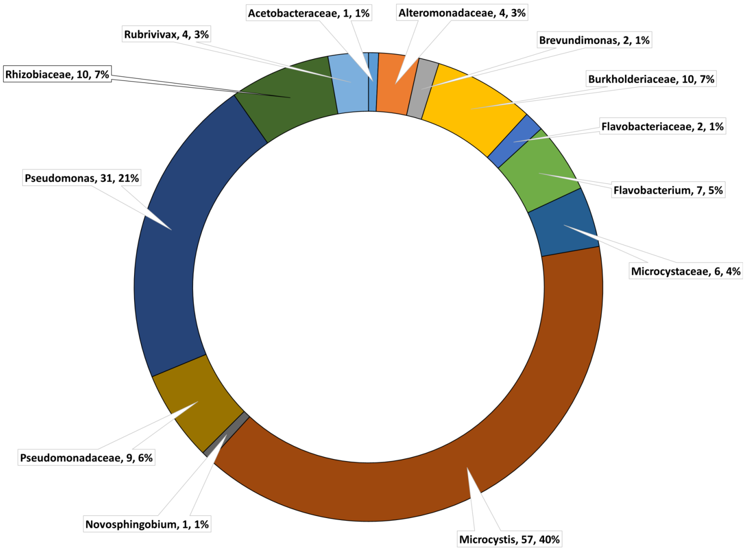

Supplementary Figure S9: **Circular plot depicting the number of features matching the Genera and the Families defined in MSnet parameters**. The percentage is from the total of 144 putatively annotated metabolites matching the taxa.

4 Metabolic modelling

Metabolic potential of associated bacteria and *Microcystis*

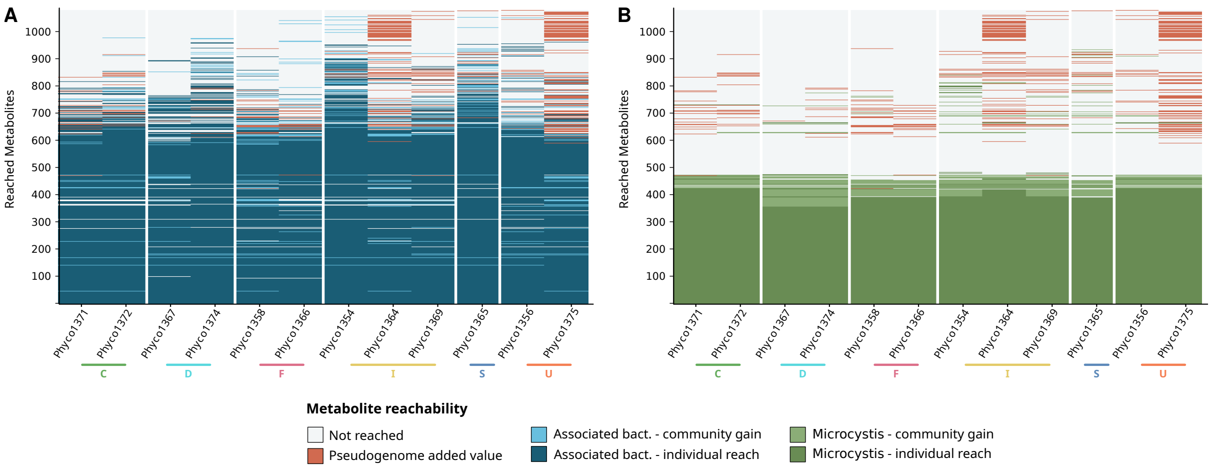

Supplementary Figure S10: **Modelling the metabolic complementarity intra-phycosphere A.** Heatmap of metabolites reached by the associated bacteria of each phycosphere grouped by genospecies (C, D, F, I, S, and U). **B.** Heatmap of metabolites reached by *Microcystis* in each phycosphere. "Individual reach" indicates that the metabolic capabilities of one member (either an associated bacterium in A, or the cyanobacterium in B) are sufficient to produce the metabolite from the growth medium. "Community gain" indicates that a member can reach the metabolites but only through metabolites exchanges, i.e. predicted cross-feeding interactions. "Not reached" depicts metabolites which are not predicted to be produced by the corresponding bacteria or cyanobacteria in the simulation, or are possibly absent from the corresponding GSMNs.

Comparison between intra-phycosphere model predictions and metabolomic signals

Comparison with merged metabolomics datasets revealed high gap between all predicted metabolites (1,078 metabolites with an InChiKey) and those actually annotated (7,696, L2-L3), as only 28 of the compounds predicted as producible could be clearly identified in the measurements. We then inspected steroids and hopanoids that were annotated through metabolomics, while their precursors, such as squalene, appeared absent. Conversely, metabolic modelling indicated that squalene can be produced in the community by certain *Microcystis* species. For the hopanoid bacteriohopanetetrol, a bacterial membrane compound annotated *in silico* in metabolomics, we identified its precursors (squalene and hopan-22(29)-ene) in the model predictions but not the final product. The presence of complete biosynthetic pathways in cyanobacterial GSMNs, along with precursors but not final products that are nevertheless detected by metabolomics suggests that modelling may preferentially reveal biosynthetic intermediates, thus partly explaining discrepancies between modelling predictions and metabolomics data.

References

[1] Méziane Aite, Marie Chevallier, Clémence Frioux, Camille Trottier, Jeanne Got, María Paz Cortés, Sebastián N. Mendoza, Grégory Carrier, Olivier Dameron, Nicolas Guillaudeux, Mauricio Latorre, Nicolás Loira, Gabriel V. Markov, Alejandro Maass, and Anne Siegel. Traceability, reproducibility and wiki-exploration for "à-la-carte" reconstructions of genome-scale metabolic models. *PLOS Computational Biology*, 14(5):e1006146, May 2018. Publisher: Public Library of Science.

[2] Philippe Bardou, Jérôme Mariette, Frédéric Escudié, Christophe Djemiel, and Christophe Klopp. jvenn: an interactive venn diagram viewer. *BMC Bioinformatics*, 15(1):293, 2014.

[3] Arnaud Belcour, Clémence Frioux, Méziane Aite, Anthony Bretaudeau, Falk Hildebrand, and Anne Siegel. Metage2Metabo, microbiota-scale metabolic complementarity for the identification of key species. *eLife*, 9:e61968, December 2020. Publisher: eLife Sciences Publications, Ltd.

[4] Haiyuan Cai, Christopher J. McLimans, Jessica E. Beyer, Lee R. Krumholz, and K. David Hambright. Microcystis pangenome reveals cryptic diversity within and across morphospecies. *Science Advances*, 9(2):eadd3783, 2023.

[5] Christiam Camacho, George Coulouris, Vahram Avagyan, Ning Ma, Jason Papadopoulos, Kevin Bealer, and Thomas L. Madden. BLAST+: architecture and applications. *BMC Bioinformatics*, 10(1):421, 2009.

[6] Ron Caspi, Richard Billington, Ingrid M Keseler, Anamika Kothari, Markus Krummenacker, Peter E Midford, Wai Kit Ong, Suzanne Paley, Pallavi Subhraveti, and Peter D Karp. The MetaCyc database of metabolic pathways and enzymes—a 2019 update. *Nucleic Acids Research*, 48(D1):D445–D453, 2019.

[7] Guntram Christiansen, Jutta Fastner, Marcel Erhard, Thomas Börner, and Elke Dittmann. Microcystin biosynthesis in planktothrix: Genes, evolution, and manipulation. 185(2):564–572. Publisher: American Society for Microbiology.

[8] The UniProt Consortium, Alex Bateman, Maria-Jesus Martin, Sandra Orchard, Michele Magrane, Aduragbemi Adesina, Shadab Ahmad, Emily H Bowler-Barnett, Hema Bye-A-Jee, David Carpentier, Paul Denny, Jun Fan, Penelope Garmiri, Leonardo Jose da Costa Gonzales, Abdulrahman Hussein, Alexandr Ignatchenko, Giuseppe Insana, Rizwan Ishtiaq, Vishal Joshi, Dushyanth Jyothi, Swaathi Kandasaamy, Antonia Lock, Aurelien Luciani, Jie Luo, Yvonne Lussi, Juan Sebastian Martinez Marin, Pedro Raposo, Daniel L Rice, Rafael Santos, Elena Speretta, James Stephenson, Prabhat Totoo, Nidhi Tyagi, Nadya Urakova, Preethi Vasudev, Kate Warner, Supun Wijerathne, Conny Wing-Heng Yu, Rossana Zaru, Alan J Bridge, Lucila Aimo, Ghislaine Argoud-Puy, Andrea H Auchincloss, Kristian B Axelsen, Parit Bansal, Delphine Baratin, Teresa M Batista Neto, Marie-Claude Blatter, Jerven T Bolleman, Emmanuel Boutet, Lionel Breuza, Blanca Cabrera Gil, Cristina Casals-Casas, Kamal Chikh Echioukh, Elisabeth Coudert, Beatrice Cuche, Edouard de Castro, Anne Estreicher, Maria L Famiglietti, Marc Feuermann, Elisabeth Gasteiger, Pascale Gaudet, Sebastien Gehant, Vivienne Gerritsen, Arnaud Gos, Nadine Gruaz, Chantal Hulo, Nevila Hyka-Nouspikel, Florence Jungo, Arnaud Kerhornou, Philippe Le Mercier, Damien Lieberherr, Patrick Masson, Anne Morgat, Salvo Paesano, Ivo Pedruzzi, Sandrine Pilbout, Lucille Pourcel, Sylvain Poux, Monica Pozzato, Manuela Pruess, Nicole Redaschi, Catherine Rivoire, Christian J A Sigrist, Karin Sonesson, Shyamala Sundaram, Anastasia Sveshnikova, Cathy H Wu, Cecilia N Arighi, Chuming Chen, Yongxing Chen, Hongzhan Huang, Kati Laiho, Minna Lehvaslaiho, Peter McGarvey, Darren A Natale, Karen Ross, C R Vinayaka, Yuqi Wang, and Jian Zhang. UniProt: the universal protein knowledgebase in 2025. *Nucleic Acids Research*, 53(D1):D609–D617, 2024.

[9] Kai Dührkop, Markus Fleischauer, Marcus Ludwig, Alexander A Aksenov, Alexey V Melnik, Marvin Meusel, Pieter C Dorrestein, Juho Rousu, and Sebastian Böcker. Sirius 4: a rapid tool for turning tandem mass spectra into metabolite structure information. *Nature methods*, 16(4):299–302, 2019.

[10] Kai Dührkop, Huibin Shen, Marvin Meusel, Juho Rousu, and Sebastian Böcker. Searching molecular structure databases with tandem mass spectra using csi:fingerid. *Proceedings of the National Academy of Sciences*, 112(41):12580–12585, 2015.

[11] Oliver Ebenhöh, Thomas Handorf, and Reinhart Heinrich. Structural analysis of expanding metabolic networks. *Genome informatics. International Conference on Genome Informatics*, 15(1):35–45, 2004.

[12] Ophélie Fraisier-Vannier, Justine Chervin, Guillaume Cabanac, Virginie Puech, Sylvie Fournier, Virginie Durand, Aurélien Amiel, Olivier André, Omar Abdelaziz Benamar, Bernard Dumas, Hiroshi Tsugawa, and Guillaume Marti. MS-CleanR: A feature-filtering workflow for untargeted LC–MS based metabolomics. *Analytical Chemistry*, 92(14):9971–9981, 2020.

[13] Pereira Francisco, Duthen, Crossay, Alignan, Hennechart, Perez, and Marti. MS-net: Multi-similarity based network annotation for untargeted metabolomics. *Research Square*, 2025.

[14] Leslie M. Hicks, Michelle C. Moffitt, Laura L. Beer, Bradley S. Moore, and Neil L. Kelleher. Structural characterization of in vitro and in vivo intermediates on the loading module of microcystin synthetase. 1(2):93–102. Publisher: American Chemical Society.

[15] Kalai Vanii Jayaseelan, Pablo Moreno, Andreas Truszkowski, Peter Ertl, and Christoph Steinbeck. Natural product-likeness score revisited: an open-source, open-data implementation. *BMC Bioinformatics*, 13(1):106, 2012.

[16] Martin R. Jones, Ernani Pinto, Mariana A. Torres, Fabiane Dörr, Hanna Mazur-Marzec, Karolina Szubert, Luciana Tartaglione, Carmela Dell'Aversano, Christopher O. Miles, Daniel G. Beach, Pearse McCarron, Kaarina Sivonen, David P. Fewer, Jouni Jokela, and Elisabeth M.-L. Janssen. CyanoMetDB, a comprehensive public database of secondary metabolites from cyanobacteria. *Water Research*, 196:117017, 2021.

[17] Martin R. Jones, Ernani Pinto, Mariana A. Torres, Fabiane Dörr, Hanna Mazur-Marzec, Karolina Szubert, Luciana Tartaglione, Carmela Dell'Aversano, Christopher O. Miles, Daniel G. Beach, Pearse McCarron, Kaarina Sivonen, David P. Fewer, Jouni Jokela, and Elisabeth M.-L. Janssen. Cyanometdb, a comprehensive public database of secondary metabolites from cyanobacteria. *Water Research*, 196:117017, 2021.

[18] Hyun Woo Kim, Mingxun Wang, Christopher A. Leber, Louis-Félix Nothias, Raphael Reher, Kyo Bin Kang, Justin J. J. van der Hooft, Pieter C. Dorrestein, William H. Gerwick, and Garrison W. Cottrell. NPClassifier: A deep neural network-based structural classification tool for natural products. *Journal of Natural Products*, 84(11):2795–2807, 2021.

[19] Marcus Ludwig, Louis-Félix Nothias, Kai Dührkop, Irina Koester, Markus Fleischauer, Martin A Hoffmann, Daniel Petras, Fernando Vargas, Mustafa Morsy, Lihini Aluwihare, et al. Database-independent molecular formula annotation using gibbs sampling through zodiac. *Nature Machine Intelligence*, 2(10):629–641, 2020.

[20] Jean-Marie Meyer, Trân Van Van, Alain Stintzi, Odile Berge, and Günther Winkelmann. Ornibactin production and transport properties in strains of burkholderia vietnamiensis and burkholderia cepacia (formerly pseudomonas cepacia). *Biometals*, 8(4):309–317, 1995.

[21] B. S. Moore. Biosynthesis of marine natural products: Microorganisms and macroalgae. *Natural Product Reports*, 16(6):653–674, December 1999.

[22] Richard E. Moore, Jian Lu Chen, Bradley S. Moore, Gregory M. L. Patterson, and Wayne W. Carmichael. Biosynthesis of microcystin-LR. Origin of the carbons in the adda and masp units. *Journal of the American Chemical Society*, 113(13):5083–5084, June 1991. Publisher: American Chemical Society.

[23] Coralie Muller, Juliette Audemard, Sylvain Prigent, and Clémence Frioux. Metanetmap: automatic mapping of metabolomic data onto metabolic networks. *bioRxiv*, 2026.

[24] Leanne A. Pearson, Kevin D. Barrow, and Brett A. Neilan. Characterization of the 2-hydroxy-acid dehydrogenase McyI, encoded within the microcystin biosynthesis gene cluster of Microcystis aeruginosa PCC7806. *The Journal of Biological Chemistry*, 282(7):4681–4692, February 2007.

[25] Kenneth L. Rinehart, Michio Namikoshi, and Byoung W. Choi. Structure and biosynthesis of toxins from blue-green algae (cyanobacteria). *Journal of Applied Phycology*, 6(2):159–176, April 1994.

[26] D. F. Robinson and L. R. Foulds. Comparison of phylogenetic trees. *Mathematical Biosciences*, 53(1):131–147, 1981.

[27] Heike Sielaff, Elke Dittmann, Nicole Tandeau De Marsac, Christiane Bouchier, Hans Von Döhren, Thomas Börner, and Torsten Schwecke. The mcyF gene of the microcystin biosynthetic gene cluster from microcystis aeruginosa encodes an aspartate racemase. 373:909–916.

[28] Inonge Noni Siziya, Chi Young Hwang, and Myung-Ji Seo. Antioxidant potential and capacity of microorganism-sourced c30 carotenoids—a review. *Antioxidants*, 11(10), 2022.

[29] Lloyd W Sumner, Alexander Amberg, Dave Barrett, Michael H Beale, Richard Beger, Clare A Daykin, Teresa W-M Fan, Oliver Fiehn, Royston Goodacre, Julian L Griffin, et al. Proposed minimum reporting standards for chemical analysis: chemical analysis working group (cawg) metabolomics standards initiative (msi). *Metabolomics*, 3(3):211–221, 2007.

[30] Daniel Tillett, Elke Dittmann, Marcel Erhard, Hans von Döhren, Thomas Börner, and Brett A Neilan. Structural organization of microcystin biosynthesis in *Microcystis aeruginosa* PCC7806: an integrated peptide–polyketide synthetase system. *Chemistry & Biology*, 7(10):753–764, October 2000.

[31] Hiroshi Tsukiura, Minoru Hanada, Kyoichiro Saito, Kei-Ichi Fujisawa, Takeo Miyaki, Hideo Koshiyama, and Hiroshi Kawagushi. Sorbistin, a new aminoglycoside antibiotic complex of bacterial origin i. production, isolation and properties. *The Journal of Antibiotics*, 29(11):1137–1146, 1976.

[32] Shuyi Zhang and Donald A. Bryant. The tricarboxylic acid cycle in cyanobacteria. 334(6062):1551–1553, 2011.

[33] Chuanqiao Zhou, Yu Peng, Miaotong Yu, Yang Deng, Li Chen, Lanqing Zhang, Xiaoguang Xu, Siyuan Zhang, Yan Yan, and Guoxiang Wang. Severe cyanobacteria accumulation potentially induces methylotrophic methane producing pathway in eutrophic lakes. *Environmental Pollution (Barking, Essex: 1987)*, 292:118443, 2022.
